# The telomere-to-telomere genome of Pucai (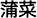) (*Typha angustifolia* L.): a distinctive semiaquatic vegetable with lignin and chlorophyll as quality characteristics

**DOI:** 10.1093/hr/uhaf079

**Published:** 2025-03-11

**Authors:** Ya-Peng Li, Li-Yao Su, Ting Huang, Hui Liu, Shan-Shan Tan, Yuan-Jie Deng, Ya-Hui Wang, Ai-Sheng Xiong

**Affiliations:** State Key Laboratory of Crop Genetics & Germplasm Enhancement and Utilization, Ministry of Agriculture and Rural Affairs Key Laboratory of Biology and Germplasm Enhancement of Horticultural Crops in East China, College of Horticulture, Nanjing Agricultural University, Nanjing, Jiangsu 210095, China; State Key Laboratory of Crop Genetics & Germplasm Enhancement and Utilization, Ministry of Agriculture and Rural Affairs Key Laboratory of Biology and Germplasm Enhancement of Horticultural Crops in East China, College of Horticulture, Nanjing Agricultural University, Nanjing, Jiangsu 210095, China; State Key Laboratory of Crop Genetics & Germplasm Enhancement and Utilization, Ministry of Agriculture and Rural Affairs Key Laboratory of Biology and Germplasm Enhancement of Horticultural Crops in East China, College of Horticulture, Nanjing Agricultural University, Nanjing, Jiangsu 210095, China; State Key Laboratory of Crop Genetics & Germplasm Enhancement and Utilization, Ministry of Agriculture and Rural Affairs Key Laboratory of Biology and Germplasm Enhancement of Horticultural Crops in East China, College of Horticulture, Nanjing Agricultural University, Nanjing, Jiangsu 210095, China; State Key Laboratory of Crop Genetics & Germplasm Enhancement and Utilization, Ministry of Agriculture and Rural Affairs Key Laboratory of Biology and Germplasm Enhancement of Horticultural Crops in East China, College of Horticulture, Nanjing Agricultural University, Nanjing, Jiangsu 210095, China; State Key Laboratory of Crop Genetics & Germplasm Enhancement and Utilization, Ministry of Agriculture and Rural Affairs Key Laboratory of Biology and Germplasm Enhancement of Horticultural Crops in East China, College of Horticulture, Nanjing Agricultural University, Nanjing, Jiangsu 210095, China; State Key Laboratory of Crop Genetics & Germplasm Enhancement and Utilization, Ministry of Agriculture and Rural Affairs Key Laboratory of Biology and Germplasm Enhancement of Horticultural Crops in East China, College of Horticulture, Nanjing Agricultural University, Nanjing, Jiangsu 210095, China; State Key Laboratory of Crop Genetics & Germplasm Enhancement and Utilization, Ministry of Agriculture and Rural Affairs Key Laboratory of Biology and Germplasm Enhancement of Horticultural Crops in East China, College of Horticulture, Nanjing Agricultural University, Nanjing, Jiangsu 210095, China

## Abstract

Pucai (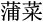) (*Typha angustifolia* L.), within the *Typha* spp., is a distinctive semiaquatic vegetable. Lignin and chlorophyll are two crucial traits and quality indicators for Pucai. In this study, we assembled a 207.00-Mb high-quality gapless genome of Pucai, telomere-to-telomere (T2T) level with a contig N50 length of 13.73 Mb. The most abundant type of repetitive sequence, comprising 16.98% of the genome, is the long terminal repeat retrotransposons (LTR-RT). A total of 30 telomeres and 15 centromeric regions were predicted. Gene families related to lignin, chlorophyll biosynthesis, and disease resistance were greatly expanded, which played important roles in the adaptation of Pucai to wetlands. The slow evolution of Pucai was indicated by the σ whole-genome duplication (WGD)-associated *Ks* peaks from different Poales and the low activity of recent LTR-RT in Pucai. Meanwhile, we found a unique WGD event in Typhaceae. A statistical analysis and annotation of genomic variations were conducted in interspecies and intraspecies of *Typha*. Based on the T2T genome, we constructed lignin and chlorophyll metabolic pathways of Pucai. Subsequently, the candidate structural genes and transcription factors that regulate lignin and chlorophyll biosynthesis were identified. The T2T genomic resources will provide molecular information for lignin and chlorophyll accumulation and help to understand genome evolution in Pucai.

## Introduction

**Figure 1:**
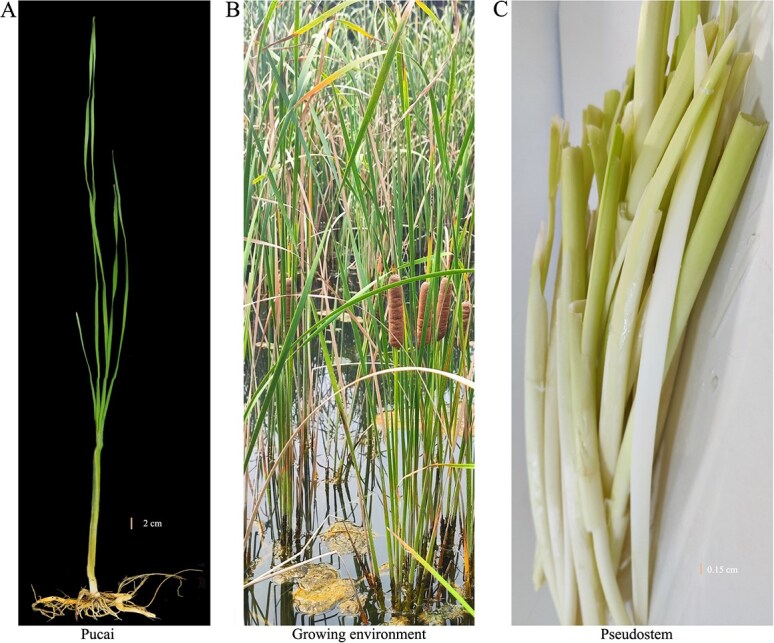
Phenotypes and growing environment of Pucai. (A): Phenotypes of Pucai. (B): Growing environment of Pucai. (C): Pseudostem of Pucai.

Pucai (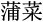) (*Typha angustifolia* L.) is a distinctive semiaquatic vegetable from Huai'an in Jiangsu, China. There is a proverb that ‘a banquet without Pucai is incomplete’ in Huai'an. It is also called ‘Kang jin cai’ (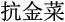). Pucai, within the *Typha* spp*.*, native to Eurasia [1, 2], is widely distributed in temperate and tropical wetlands across the northern and southern hemispheres [1, 3, 4] (Fig. 1A)*.* Pucai is cultivated primarily in marshes and can be utilized as an ornamental plant (Fig. 1B). The leaves of Pucai have been used in the fabrication of textiles*.* Its dried pollen is known as 'Pollen Typhae' and has medicinal value [5]. The pseudostem of Pucai is encased in leaf sheaths and harvested as an edible vegetable in autumn (Fig. 1C). The pseudostem is full of nutrients like protein, carbs, and vitamins. It can help to keep blood sugar steady, soothe the heart and lungs, and support the spleen and stomach. Its palatable edible components, nutritional value, and capacity for long-term storage make it commercially attractive on a large scale*.*

Lignin and chlorophyll represent two crucial traits and quality indicators for Pucai. To provide a theoretical basis for cultivating high-quality Pucai, the focus was directed to the lignin and chlorophyll biosynthesis of Pucai. However, the pseudostem in shallow water displays a high level of lignin content, which results in a gritty texture that affects mastication [6]. Lignin, one of the most abundant biopolymers on earth [7], is synthesized through the phenylpropanoid pathway in plants [8]. Lignin biosynthesis is a complex network comprising three stages: the synthesis of lignin monomers, subsequent transport, and the final polymerization [9]. Several key enzymes involved in lignin biosynthesis are of particular importance, including phenylalanine ammonia-lyase (PAL), CoA ligase (4CL), cinnamoyl CoA reductase (CCR), hydroxycinnamoyl-CoA shikimate (HCT), caffeic acid-*O*-methyltransferase (COMT), and ferulate 5-hydroxylase (F5H) [7, 10, 11]. Lignin monomers are synthesized within the cytoplasm and transported to the apoplast [12]. Finally, lignin is typically polymerized with monolignols by peroxidase (PER) and laccase (LAC) in the secondary cell wall [13]. Transcriptional regulation by NAC, MYB, AP2/ERF, TCP transcription factors (TFs), and additional TFs is believed to be a pivotal regulatory mechanism for lignin biosynthesis and has been extensively investigated [14–18].

Meanwhile, the accumulation of chlorophyll negatively impacts appearance and consumer satisfaction. Chlorophyll biosynthesis in plants can be divided into three distinct phases. The first phase involves the synthesis of chlorophyll *a* from glutamate [19–21]. The second phase covers the interconversion between chlorophyll *a* and chlorophyll *b*, which is also known as the chlorophyll cycle [22]. The final phase of chlorophyll metabolism entails the degradation of chlorophyll *a* [20, 23]. Most of the enzymes involved in chlorophyll metabolism have been identified, including chlorophyll *a* oxygenase (CAO), chlorophyll *a* synthase (CHLG), chlorophyllide *b* reductase (NOL/NYC1), pheophorbide *a* oxygenase (PAO), divinyl chlorophyllide *a* 8-vinyl-reductase (DVR), and others [22, 24–26]. TFs such as MYB, bZIP, and GLK have been reported to regulate the accumulation of chlorophyll [27–29].

High-quality genomes serve as powerful molecular tools offering deeper insights into plant trait genetics [30], including the mechanisms of lignin and chlorophyll biosynthesis in the Pucai. In 2022, the first genome of *Typha*, *Typha latifolia*, was sequenced and assembled from PacBio long-read and high-coverage Illumina sequences [31]. The N50 contig length for the genome assembly of *T. angustifolia* was improved, but there are still many unknown regions [5]. The gapless assemblies for the model crop of Poales [32, 33], the horticultural crop [34–36], and the vegetable [37, 38] were recently generated with the advances in PacBio HiFi reads. Telomere-to-telomere (T2T) assembly denotes the construction of continuous sequences encompassing entire chromosomes from one telomere to the opposing telomere, which facilitates studies on plant genetics and enables more accurate chromosome maps [39]. Despite these advancements, much of the current research on Pucai focuses on cultivation and environmental remediation, which falls short of fully addressing the needs of Pucai research. To obtain the T2T genome of Pucai, a sequencing analysis was performed on a diploid Pucai belonging to the Huai'an variety. Combining data from Oxford Nanopore Technology (ONT) Ultra-long and PacBio High-fidelity (HiFi) sequencing techniques were integrated with high-throughput chromosome conformation capture (Hi-C) to construct the T2T Pucai genome. This approach resulted in a comprehensive Pucai genome characterized by high continuity, integrity, and accuracy. Subsequent gene annotation and comparative genomic analyses were conducted. Furthermore, candidate structural genes and TFs implicated in the biosynthesis of lignin and chlorophyll in Pucai were identified. The results of this study address previously uncharacterized regions of the Pucai genome, thereby establishing a genetic foundation for prospective molecular breeding on Pucai.

## Results

### Genome assembly and gene annotation of the TaT2T genome

**Figure 2:**
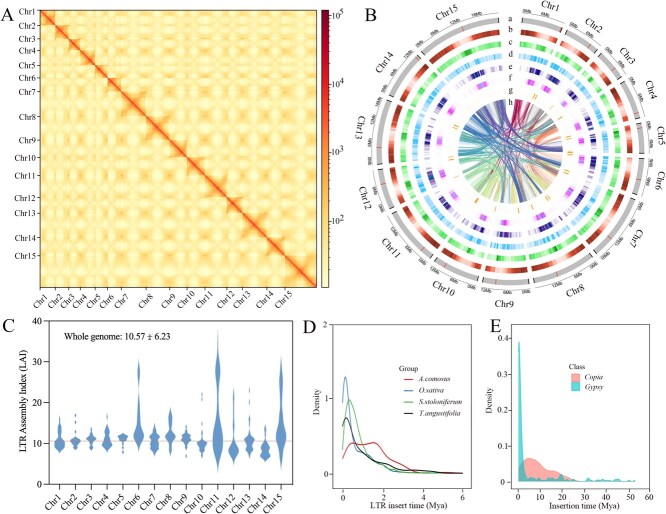
T2T complete reference genome of Pucai. (A): Hi-C interaction matrix based on the TaT2T assembly. (B): Circus display of significant characteristics of the TaT2T genome. (a), chromosome names and sizes, with centromere (red) and telomere (black) positions marked (b), density of HC genes (c), distribution of GC content (d), density of DNA TE (e), density of *Copia* (f), density of *Gypsy* (g), density of satellite repeats (h), links between syntenic genes. (C): LAI of the TaT2T genome. (D): LTR-RT insertion bursts of Pucai compared to those in *O. sativa*, *S. stoloniferum,* and *A. comosus*. (E): Insertion bursts of *Gypsy* and *Copia* of Pucai.

To preliminarily characterize the genomic features of Pucai, next-generation sequencing and *K*-mer analysis were conducted to estimate the genome size and heterozygosity. The *K*-mer frequency distribution (*k* = 19) indicated that the genome size of Pucai is ~228.08 Mb, with a heterozygosity rate of 0.43% (Fig. S1 and Table S1). Following the application of ONT Ultra-long, PacBio HiFi, next-generation, and Hi-C sequencing techniques, the resulting pass reads were 23.14, 25.44, 22.81, and 46.95 Gb, respectively (Tables S2–S5). The initial assembly of the genome, which utilized the original ONT Ultra-long and HiFi data, revealed four gaps. By comparing the hole-filling data (without N) with the genomic gap region (with N), the four gaps were filled. The reliability of the patched gaps was verified by comparing the replaced regions in the genome after gap filling with ONT reads/HiFi reads. (Fig. S2). Ultimately, a complete T2T-level genome, devoid of gaps across 15 chromosomes, was constructed after addressing all remaining gaps. This version of the Pucai assembly was named TaT2T. The GC content of the TaT2T genome was determined to be 37.69%, with a contig N50 of 13.73 Mb. A total sequence length of 207.00 Mb encompassed all 15 chromosomes (Table 1). The Hi-C heat map corresponding to the final assembled TaT2T genome chromosomes exhibited a high intensity of interactions within each chromosome, with no anomalous interaction signals detected (Fig. 2A). In plants, telomeres are typically composed of a short repetitive sequence that is reiterated several hundred times, a structure produced by telomerase [40]. Utilizing the plant-specific seven-base telomere repeat sequence (3′-TTTAGGG/5'-CCCTAAA) as a query [34], all 30 telomeric regions were identified across the 15 chromosomes (Fig. 2B and Table S6).

**Table 1:** TaT2T genome assembly statistics.

**Genomic feature**	**Value**
Assembled genome size (Mb)	207.00
Contig N50 (Mb)	13.73
Number of scaffolds	479
Gaps	0
Number of telomeres	30
Number of predicted centromeres	15
GC content (%)	37.69
BUSCOs (%)	96.9
QV LAI	42.85 10.57

Based on *de novo*, homology predictions, and transcriptome (next-generation and third-generation transcriptome), 22 427 genes, 128 843 exons, and 106 416 introns were identified in the TaT2T genome (Table S7). The completeness of the gene prediction was evaluated using benchmarking universal single-copy orthologs (BUSCO), resulting in the identification of 97.50% of the complete set of BUSCO genes in the TaT2T genome. (Table S8). The average lengths of mRNA, coding sequence (CDS), exon, and intron were 4944.65, 1268.27, 311.2, and 662.68 bp, respectively. The length distribution of genes, CDS, exons, and introns in the TaT2T genome was compared with those of *Ananas comosus*, *Arabidopsis thaliana*, *Rhynchospora tenuis*, *Juncus effusus*, and *Sparganium stoloniferum* (Fig. S3). The results revealed that the frequency distribution of gene and intron lengths within the TaT2T genome exhibited similarities to that observed in other species. A variety of protein databases were employed to conduct a comparative analysis of the protein sequences encoded by the predicted genes. Among these, 21 525 genes, representing 95.98%, were matched to the database and received at least one form of annotation (Table S9). A total of 9787 genes were annotated in the kyoto encyclopedia of genes and genomes (KEGG) database, 20 782 in the Nr database, 20 765 in UniProt, 11 816 in gene ontology (GO), 168 in KOG, 16 964 in Pfam, and 21 309 in the InterPro public databases (Fig. S4).

Furthermore, the noncoding RNAs of the TaT2T genome were identified. A total of 372 transfer RNAs (tRNAs), encompassing a cumulative length of 27 896 bp, were annotated based on structural features. An analysis utilizing Rfam and other ribosomal RNA (rRNA) databases revealed the presence of 209 micro RNAs (miRNAs), with an average length of 121 bp within the TaT2T genome. In addition, 207 and 236 different types of rRNAs and small nuclear RNAs (snRNAs) were annotated (Table S10).

To assess genomic consistency, the reads generated through next-generation sequencing were aligned with the assembled TaT2T genome. The mapping rate reached 98.5% with a coverage rate of 99.82% (Table S11). A BUSCO assessment demonstrated that 96.9% of the conserved genes were matched, comprising 1543 single-copy homologous genes and 21 multicopy homologous genes (Table S12). The quality value (QV) of the TaT2T genome was established at 42.85 (Table 1), with the QV value of each chromosome falling between 38.73 and 46.40 (Table S13). Furthermore, the integrity and continuity of the assembly were confirmed by evaluating the completeness of long-terminal repeat (LTR) retrotransposons through the LTR Assembly Index (LAI). LAI index >10 is suggested as a threshold to meet the reference quality genomes [41]. The TaT2T genome provided an LAI value of 10.57 (Fig. 2C). Collectively, the results illustrated the high quality, reliability, and accuracy of the TaT2T genome assembly.

### Analysis of TEs in the TaT2Tgenome

In plants, bursts of repetitive sequences are one of the principal responsible for genome expansion [42]. The representative genome of the monocot plants has more abundance of repetitive elements. The majority of the repetitive elements in the *Acorus* genome (38.40%) are comprised of LTRs [43], likewise, Schott also has 42.12% repetitive regions majorly composed of the LTR-RTs *Gypsy* subfamilies [44]. Following the combination of the prediction results and the elimination of redundancy, 77.32 Mb of duplicated sequences were obtained, representing 37.35% of the TaT2T genome. The LTR-RT is the most abundant type of repetitive sequence, spanning 16.98% of the genome and numbering 101 218. The *Gypsy* and *Copia* comprise ~11.91% and ~ 4.04% of the TaT2T genome, respectively (Table S14). The second most abundant transposable element (TE) was DNA transposons, comprising ~2.21% of the TaT2T genome. The remaining interspersed repeats, comprising LINEs (Long Interspersed Nuclear Elements) and SINEs (Short Interspersed Nuclear Elements), account for 1.74% and 0.01% of the TaT2T genome, respectively (Table S14). Meanwhile, identified tandem repeats (satellite repeats) span 0.23% of the TaT2T genome (Table S14). A circus map of the TaT2T genome was constructed for the visualization of important features (Fig. 2B).

A distinctive unimodal distribution was evident for the insertion times of intact LTR-RTs in *Oryza sativa*, *S. stoloniferum*, and *T. angustifolia*. In contrast, a bimodal unimodal distribution was observed for *A. comosus.* The density of recent LTR-RT insertions in *T. angustifolia* was relatively low, with the peak of amplification appearing ~0.25 million years ago (Mya). This was the most recent among the four species (Fig. 2D). At the superfamily level, a recent burst of *Gypsy* elements in *T. angustifolia* was found at ~0.3 Mya. Meanwhile, amplifications of *Gypsy* retrotransposons dominantly shaped the unimodal distribution pattern of LTR-RT burst dynamics (Fig. 2E).

### Identification of candidate centromeres in the TaT2Tgenome

Centromeres are defined as specific regions on dividing chromosomes to which spindle microtubules bind. Plant centromeres typically consist of tandemly repeated monomers. Till now, the centromere structures of Pucai remain unclear. The TaT2T assemblies facilitated the examination of these regions. First, tandem repeats were identified along the chromosomes. Based on the sequence characteristics of a continuous high density of short tandem repeats and a low density of genes, centromeric regions were predicted. The approximate locations of 15 centromeric regions were estimated, with lengths ranging from 1.16 to 215.76 Kb (Fig. 2B and Table S6). Notably, some centromeres were positioned at the terminal regions of the chromosome.

### Orthologous clustering and phylogenetic status of Pucai

**Figure 3:**
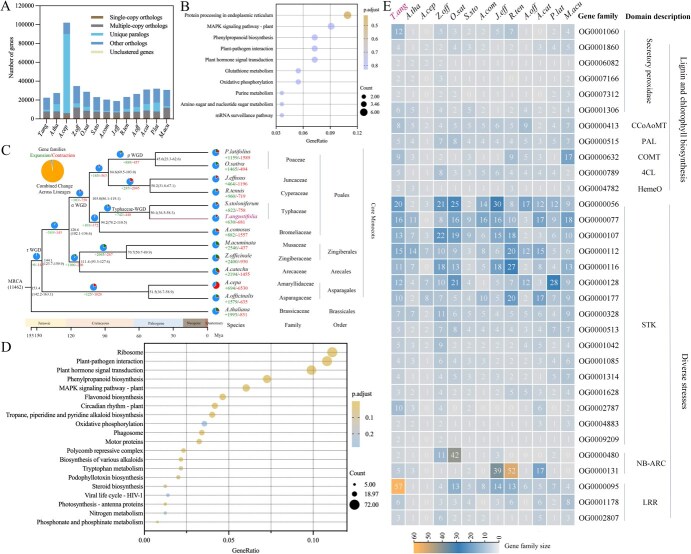
Comparative genomics of gene families of Pucai. (A): Number of shared orthologous gene families among 13 genomes. (B): KEGG enrichment of Pucai-unique gene families. (C): Estimation of divergence time and expanded/contracted gene families. (D): KEGG enrichment of expanded gene families of Pucai. (E): Functional annotation for the expanded gene families in Pucai. The left panel illustrates the sizes of gene families across 13 species, while the right panel presents the functional annotations associated with these gene families.

To uncover the genomic basis of distinctive phenotypes of Pucai, an analysis was conducted on the evolution of gene families by examining both unique and shared gene families across various plant species. A total of 13 species genomes were selected for the identification of homologous genes, the execution of gene family clustering analyses, and the enrichment of single-copy and multiple-copy genes (Fig. 3A). A total of 77 411 orthologous gene families were identified across all species, which included 421 530 genes. Among these, 3831 gene families were determined to be shared by all species, accounting for 108 175 genes. A total of 319 gene families were classified as single-copy gene families. Additionally, the TaT2T genome comprises 1604 unique gene families, which encompass 1928 unique paralogs. Forty-two of these unique paralogs contain PKc-like superfamily domain, a signaling motif shared the calcium-phospholipid-dependent kinases, implying that a stronger signaling system to sense the external environment had evolved in Pucai. GO enrichment of the unique gene families of Pucai was mainly related to protein kinase activity and protein serine/threonine kinase activity (Fig. S5). KEGG enrichment of the unique gene families was primarily linked to the protein processing in the endoplasmic reticulum, MAPK signaling pathway-plant, phenylpropanoid biosynthesis, and plant–pathogen interaction (Fig. 3B). It is noteworthy that four unique genes are members of the PER and hydroxycinnamoyltransferase (HCT) gene families, which are associated with lignin synthesis.

To analyze the phylogenetic status of the TaT2T genome, a phylogenetic tree was generated to estimate the divergence time of the 13 species, including *A. thaliana* as the outgroup species (Fig. 3C). The Bromeliaceae, Rapateaceae, and Typhaceae comprise the early diverging grade of Poales. The divergence of lineages leading to the Poales and *A. comosus* (Bromeliaceae) within the Poales is estimated to have occurred between 95 and 115 Mya [45, 46]. The earliest *Typha* spp. and *Sparganium* L. (the other genus of Typhaceae) fossil records that have been found were from the Late Cretaceous [1]. Consistent with our analyses, Typhaceae plants were diverged from lettuce in Poales at ~94.5 Mya. In this study, divergence time analyses indicated that the *T. angustifolia* lineage originated ~54.2 Mya. An estimated 11 462 gene families were found in the common ancestor of the selected species (Fig. 3C). In the lineage leading to Typhaceae, 788 gene families were expanded, whereas 1087 gene families were contracted. Moreover, 681 gene families were found to contract, while 630 gene families expanded in Pucai. The most conspicuous contraction gene families of Pucai were mainly linked to plant hormone signal transduction (Figs S6 and S7). The expanded gene families might provide insights into the adaptability of Pucai to the environment. The KEGG enrichment analysis of the expanded gene families showed that plant–pathogen interaction and phenylpropane biosynthesis were present in the enrichment results (Fig. 3D). The functional annotations of these expanded gene families related to lignin synthesis, chlorophyll synthesis, and diverse stresses (Fig. 3E).

### Whole-genome duplications of Pucai

**Figure 4:**
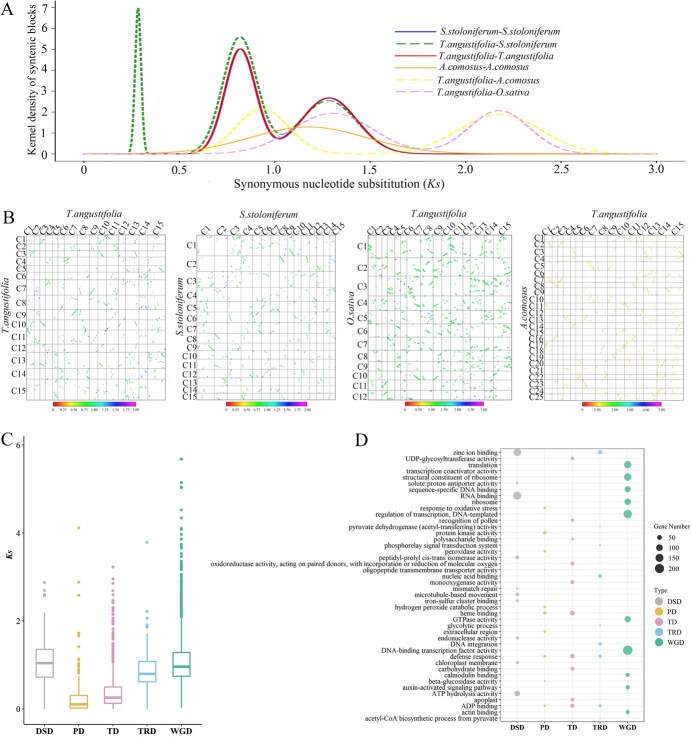
WGDs in Pucai. (A): *Ks* distribution. (B): Dot plots of paralogues and orthologues. (C): *Ks* values of gene pairs originating from WGD genes. (D): GO enrichment analyses of WGD genes.

Whole-genome duplications (WGDs) are rampant during monocot diversification [47, 48], which have been proposed as a key mechanism driving species diversification and adaptation [49]. The τ WGD was shared by most monocots with an occurrence of ~150 Mya [48]. The identification of τ WGD is a challenge due to the high level of degeneration of phylogenetic signals and lack of proper outgroups [47, 50]. The σ WGD was shared by all Poales at ~110 Mya [47, 51, 52]. The ρ WGD was shared by all Poaceae and is supposed to have occurred ~70 Mya [53]. To assess the WGD history of Pucai, we plotted the distributions of synonymous substitutions per synonymous site (*Ks*) for paralogous genes found within the genomes of Pucai and the other three Poales plants, as well as for orthologous genes (Fig. 4A). Two clear peaks at the *Ks* value of 0.82 and 1.28 for paralogs in collinear regions were shown by Pucai. Similar patterns with two peak values of 0.82 and 1.28 were also observed for *S. stoloniferum*, pointing to two possible common WGD events. Meanwhile, two distinct peaks were observed for paralogs in collinear regions in *O. sativa*. However, only one peak appeared for paralogs in *A. comosus*. A comparison of the *T. angustifolia Ks* peak of WGD with *T. angustifolia*–*A. comosus* and *T. angustifolia*–*O. sativa* indicated that this σ WGD event at a peak of ~2.17 antedated the segregation between *T. angustifolia* and *A. comosus*. It is therefore proposed that a WGD event was unique to the Typhaceae at a peak *Ks* of 0.82. The unique WGD event was defined as Typhaceae-WGD. Typhaceae experienced a σ WGD event with a peak *Ks* of ~1.20.

Furthermore, the WGD events in Pucai were also evident through both the intra-and intergenomic synteny analyses. The dot plot analysis of the Pucai genome revealed significant collinearity located in syntenic blocks (Fig. 4B). Intergenomic comparisons between *T. angustifolia* and *O. sativa* with *T. angustifolia* and *A. comosus* show common σ WGD events in Poales. An intergenomic comparison between *T. angustifolia* and *S. stoloniferum* showed common σ WGD and Typhaceae-WGD events.

Gene duplication has been regarded as a major force for evolution [54]. In the Pucai genome, 14 824 duplication genes were classified into five categories: 6731 (45.41%) dispersed duplication genes (DSD), 5532 (37.32%) whole-genome duplicates (WGD) genes, 820 (5.53%) tandem duplication genes (TD), 347 (2.34%) proximal duplication genes (PD), 1394 (9.40%) transposed duplication genes (TRD). The discovery of smaller *Ks* values for TD and PD gene pairs provides evidence of recent TD and PD events (Fig. 4C). It is noteworthy that duplications across all five modes were associated with distinct enriched GO terms (Fig. 4D). For example, WGD genes exhibited significant enrichment in categories related to DNA-binding TF activity. The categories enriched for genes resulting from PD were associated with responses to oxidative stress and hydrogen peroxide, whereas the TD genes were linked to defense responses. KEGG analysis revealed that PD and TD genes were mainly enriched in plant–pathogen interaction and secondary metabolic pathways (Figs S8 and S9). These results suggest that recent PD and TD events may benefit the adaptation of Pucai to the pathogen diversity of wetlands.

### Genomic variation among *Typha* genomes

**Figure 5:**
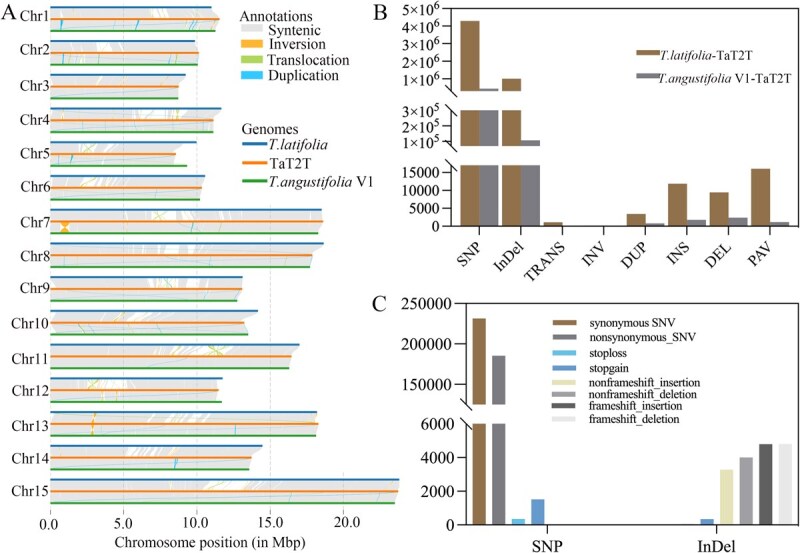
Huge number of variations among *Typha* genomes. (A): Syntenic analyses among the *T. latifolia*, *T. angustifolia* V1, and TaT2T genomes. (B): Statistics of inter- and intraspecies variations. (C): Annotation of interspecies SNPs and InDel.

The divergence observed within the genus *Typha* spp. suggested significant genomic variability among the *Typha* genomes. In this study, we conducted a comparative analysis of two previously published *Typha* assemblies (*T. latifolia* [31] and *T. angustifolia* V1 [5]) alongside a newly assembled TaT2T genome (Fig. 5A). Our results indicated that the genomic variations are more pronounced between species than within species. Specifically, an average of 45.78 Mb (22.19%) of genomic regions exhibited alignment challenges across different species due to a high density of variants, which is ~37.83 times greater than the average of 1.21 Mb observed in intraspecies comparisons. Furthermore, the frequency of interspecies single nucleotide polymorphism (SNPs), InDels, structure variantions (SVs), and presence-absence variation (PAVs) in syntenic regions surpassed that of intraspecies variations (Fig. 5B). Among the five SV types, deletions (DELs) and insertions (INSs) were the most abundant. These variations led to numerous discrepancies in gene structures and locations, resulting in genome-wide differences in both coding and intergenic regions.

Subsequently, the functions of interspecies SNPs and InDel were annotated. It was determined that the functions of the SNPs are primarily synonymous SNV and nonsynonymous SNV, with a few stoploss and stopgain variants. Nevertheless, the functions of indel are primarily nonframeshift insertion, nonframeshift deletion, frameshift insertion, and frameshift deletion (Fig. 5C). The enrichment of SVs and PAV between species indicated that both were predominantly enriched in hydrolase activity and carbon metabolism (Figs S10–S13).

### Lignin and chlorophyll biosynthesis of Pucai

**Figure 6:**
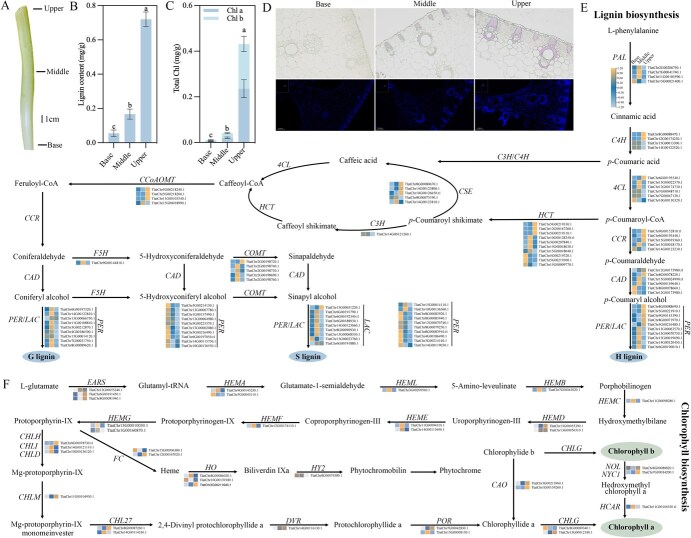
Lignin and chlorophyll content and biosynthesis of Pucai. (A): Three sampling sections of the Pucai pseudostem. (B): Lignin content. (C): Chlorophyll content. (D): Cross-section after treatment with phloroglucin-HCL and fluorescence micrographs. (E): Expression profiles of genes involved in lignin biosynthesis. (F): Expression profiles of genes involved in chlorophyll biosynthesis.

The pseudostem was divided into three sections, designated as upper, middle, and base (Fig. 6A). The three sections were subjected to examination to ascertain lignin and chlorophyll contents. The results demonstrated a progressive elevation in lignin concentration across the base, middle, and upper regions. Notably, the lignin content of the upper region reached 0.72 mg/g, exhibiting a 13.10- and 4.32-fold increase compared to the base and middle regions, respectively (Fig. 6B). The additional identification of lignin accumulation in the samples from the various treatments using phloroglucin-HCL staining demonstrated that this was by the results of the lignin content determination described above (Fig. 6D). Furthermore, the accumulation of lignin was predominantly observed in the xylem. The results of the autofluorescence analyses indicated that the vascular bundles in the base part were not fully developed and were relatively scarce in number (Fig. 6D). In the middle and upper parts, although the number of vascular bundles remained unchanged, the area of the xylem within the vascular bundles of the upper part was greater than that of the middle part and extended toward the formation layer. Consequently, the lignin content deposited in the xylem conduits was observed to increase in the base, middle, and upper parts. The trend in chlorophyll content was analogous to that observed in lignin content. The total chlorophyll content of the upper region demonstrated a 187.50- and 20.56-fold increase in comparison to the base and middle regions, respectively (Fig. 6C).

Subsequently, lignin and chlorophyll biosynthesis of Pucai were constructed based on the TaT2T genomic data. A total of 99 genes were identified as structural genes in lignin biosynthesis and 39 as structural genes in chlorophyll biosynthesis through a process of homology comparison and screening. The structural genes involved in lignin biosynthesis were distributed across the 15 chromosomes of Pucai. Most of these genes were concentrated on chromosomes 15, 8, 14, and 5, with 14, 13, 10, and 10 genes, respectively (Fig. S14). Except for *F5H*, which is a single-copy gene, other genes involved in lignin biosynthesis have multiple copies located on the same or different chromosomes. The *PER* and *HTC* genes were found to have 39 and 10 copies, respectively. Tandem gene duplications seem to have contributed to the increase in the number of structural genes in lignin biosynthesis. The distribution of various gene families in close on chromosomes may prove advantageous for the enhancement of lignin biosynthesis efficiency. Subsequently, an examination of the expression of structural genes involved in lignin biosynthesis was conducted through RNA sequencing (RNA-seq) analyses (Fig. 6E). The results demonstrated that the expression of *CCR* orthologs (TlatChr15G00019360.1), *COMT* orthologs (TlatChr2G00198720.1), *CSE* orthologs (TlatChr8G00080670.1 and TlatChr14G00123800.1), *HTC* orthologs (TlatChr5G00219330.1, TlatChr12G00167260.1, and TlatChr5G00219310.1), and *PER* orthologs (TlatChr8G00088690.1, TlatChr5G00221910.1, TlatChr5G00216480.1, and TlatChr8G00083910.t1) was highly induced in the upper portion of the pseudostem. The expression of these genes exhibited an inverse trend in the base portion, followed by the middle portion.

Similarly, an analysis was conducted on the structural genes involved in chlorophyll biosynthesis. The structural genes were predominantly distributed across the 12 chromosomes of Pucai, with a notable concentration on chromosomes 15, 8, 14, and 7 (Fig. S15). It can be observed that the *HEML*, *HEMB*, *HEMC*, *HEMF*, *CHLM*, *HY2*, *DVR*, and *HCAR* genes are single copies. The expression levels of TlatChr11G00104930.1 (*CHLM*), TlatChr15G00000690.1 (*CLH*), TlatChr12G00167020.1 (*FC*), TlatChr7G00034200.1 (*NOL*), TlatChr8G00082810.1 (*NYC1*), and TlatChr8G00087260.1 (*CHLE*) were found to increase sequentially in the base, middle, and upper based on the expression pattern of the genes involved in chlorophyll biosynthesis (Fig. 6F).

### Potential key genes involved in lignin and chlorophyll biosynthesis of Pucai

**Figure 7:**
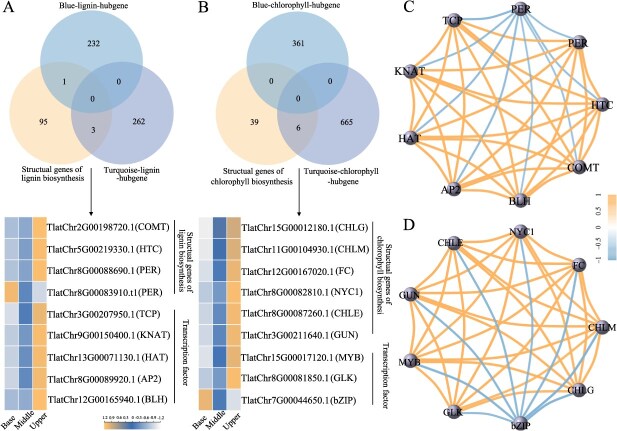
Potential key genes involved in lignin and chlorophyll biosynthesis. (A): Venn map and heat map of key genes involved in lignin biosynthesis. (B): Venn map and heat map of key genes in chlorophyll biosynthesis. (C): Network diagram of key genes involved in lignin biosynthesis. (D): Network diagram of key genes involved in chlorophyll biosynthesis.

To investigate coexpression networks, the WGCNA (Weighted Gene Coexpression Network Analysis) was employed based on transcriptome data. As illustrated in Fig. S16A, the genes were classified into four coexpression modules. A correlation was identified between gene modules and lignin and chlorophyll content, whereby the MEturquoise module exhibited a positive correlation with lignin and chlorophyll content, while the MEblue module displayed a negative correlation. (Fig. S16B). The heat maps of the genes indicated that the MEturquoise module exhibits a sequential increase in gene expression at the base, middle, and upper, whereas the MEblue module demonstrates the opposite trend (Fig. S16C). A total of 262 and 232 hub genes in lignin biosynthesis were identified through screening in the MEturquoise and MEblue modules, respectively. Consequently, four structural genes (*COMT*, *HTC*, and *PER*) and five TFs (*TCP*, *KNAT*, *HAT*, *AP2*, and *BLH*) associated with lignin biosynthesis were identified as potential key genes (Fig. 7A). Similarly, the objective was to identify six structural genes (*CHLG*, *CHLM*, *FC*, *NYC1*, *GUN*, and *CHLE*) and three TFs (*MYB*, *GLK*, *bZIP*) involved in chlorophyll biosynthesis (Fig. 7B). The Pearson correlation between potential key genes associated with lignin and chlorophyll biosynthesis was visualized (Fig. 7C and D).

## Discussion

Pucai is an important semiaquatic vegetable characterized by its pseudostem, which serves as a rich source of various vitamins and metabolites. Lignin and chlorophyll content in the pseudostem are key traits in the quality of Pucai. The assembly of T2T genomes from a range of horticultural species has facilitated the identification of essential traits [55]. For instance, the genome of bitter melon has enabled the exploration of the molecular mechanisms governing ripening and flavor development during fruit maturation [56]. Additionally, the regulatory mechanisms underlying color variation in azaleas have been elucidated through the integration of a gapless genome and multiomics approaches [57]. Nevertheless, the scarcity of high-quality genome sequences for Pucai presents a challenge that needs to be addressed. To enhance the understanding of Pucai's evolutionary patterns and genetic diversity, a comprehensive genome sequence of Pucai has been mapped. The generation of TaT2T gap-free genome assemblies marks a significant advancement in the genomic research of Pucai.

In this study, the construction of the 15 chromosomes of Pucai was achieved through the optimization of various methodologies, resulting in a genome size of 207.00 Mb. The assembled TaT2T genome, characterized by a contig N50 of 13.75 Mb, demonstrates high quality as assessed by the LAI index, mapping of Illumina reads, and BUSCO analysis. The implementation of the TaT2T gapless genome facilitated a deeper understanding of complex genomic regions, including centromeres and telomeres. Centromeres have been the subject of extensive investigation in the T2T genomes of species such as humans [58], Arabidopsis [59], and rice [60], all of which exhibit a significant presence of high-copy tandem repeats. Similarly, the centromeres of Pucai are characterized by a richness in such satellite sequences. However, experimental methods such as CENH3 ChIP-Seq and FISH are required for validation if more accurate centromere results are to be obtained. In contrast to centromeres, most telomeres also exhibit a high density of tandem telomeric repeats. The results provided a foundational theoretical framework for molecular marker-assisted breeding and the exploration of genetic functions in Pucai.

Pucai is an ancient species with estimated crown ages of 22.64–57.60 Mya [1], which matches our results originating ~54.20 Mya. However, the low activity of recent LTR-RT is related to the slow evolution [61] of Pucai. The nonoverlapped WGD peaks could be explained by varied mutation rates among species [53]. The σ WGD-associated *Ks* peaks from different Poales are substantially different, with those of *O. sativa* and *A. comosus* at *Ks* = 2.17, and those of *T. angustifolia* and *S. stoloniferum* at *Ks* = 1.28. These values suggested that starting from the σ WGD, genes in *T. angustifolia* and *S. stoloniferum* have evolved slower than those in *O. sativa* and *A. comosus*. At the same time, the Typhaceae-WGD event was identified in Typhaceae. Typhaceae-WGD provides further insight into the evolution of Typhaceae genomes following WGD. The results of the enrichment analyses of unique and expansion gene families imply that lignin as a physical barrier increased resistance and immune responses to pathogens and might play important roles in the adaptation of Pucai to wetland growth niches. Meanwhile, recent TD and DSD events in Pucai were related to plant defense response. Therefore, the genes associated with plant resistance, in addition to lignin and chlorophyll, may also play a pivotal role in the adaptation to environment and evolution of Pucai.

The genus *Typha* spp. encompasses ~10–15 species, each exhibiting a range of genetic characteristics and traits [1]. However, reference genomes have been established for only two of these species to date. The breadth of the elongated, linear leaves differs among the species; specifically, *T. latifolia* possesses the broadest leaves, while *T. angustifolia* features the narrowest [62]. In certain areas where their ranges overlap, *T. latifolia* and *T. angustifolia* can hybridize, resulting in the formation of the hybrid Typha×glauca [62]. Statistical analysis and annotation of SNPs and InDels were conducted between *T. latifolia* and *T. angustifolia* in this study. With more complete *Typha* spp genomes available, these results will be useful in hybridization and genetic diversity of *Typha* spp at the genomic level. SVs have been reported to be beneficial to plants, e.g. involved in biotic stresses, abiotic stresses, and local adaptation [63]. The SVs identified in the chromosomal-level phased genome may have potential implications in gene expression and function analysis in Pucai. Pucai has a large potential for removing heavy metals [64]. It is postulated that the enrichment of SVs in hydrolase activity and carbon metabolism may be a pertinent factor in this process. The genomic variation of Pucai provided important insights into the genetic diversity, agronomic traits, and candidate genes applicable for Pucai improvement.

Understanding the synthesis and genetic regulation of lignin and chlorophyll is of great value for the improvement of vegetable quality in Pucai. The lignin and chlorophyll metabolic pathways in Pucai were constructed in this study. Numerous structural genes were identified as having multiple copies, which may serve functionally redundant roles. Through RNA-seq and WGCNA analysis, nine candidate genes related to lignin biosynthesis and regulation were selected. Among them, the structural gene *COMT* is necessary for lignin biosynthesis [9], and the reduction in lignin results from *COMT* suppression in switchgrass [65]. HCT is the gateway enzyme of lignin biosynthesis [66] and downregulation of *HCT* affects lignin synthesis in Arabidopsis [67]. In addition, the accumulation of lignin within the seed coats of transgenic *Cleome* plants is significantly suppressed due to the inhibition of *PER* gene expression [68]. Six candidate TFs were identified, including *TCP*, *KNAT*, *HAT*, *AP2*, and *BLH*. Similarly, in chlorophyll synthesis and regulation, a total of eight candidate genes were identified. One such gene is the TF GLK, which belongs to the Golden2, ARR-B, and GARP TF family [69]. *GLK* has been studied in Arabidopsis, rice, tomato, and many other species, affecting chloroplast development and chlorophyll accumulation [69–71]. Another gene is *GUN*, involved in the synthesis of chlorophyll [72]. The repression of nuclear transcription of *GLK* is mediated by *GUN* [73]. Although the aforementioned literature provides evidence for a potential correlation between these candidate genes and the biosynthesis and regulation of lignin and chlorophyll, further experimentation is required to elucidate the function in the Pucai.

In this research, we generated a T2T gap-free genome for Pucai, accompanied by high-quality assembly, annotation, and analysis. Phylogenomic and multiomic approaches utilizing the TaT2T genome elucidated the mechanisms responsible for the distinctive and tissue-specific accumulation of lignin and chlorophyll. The resources from the TaT2T genome will serve as a significant reference for future investigations in monocot genomics and biology, thereby facilitating research advancements and fostering targeted improvements in Pucai.

## Conclusion

**Figure 8:**
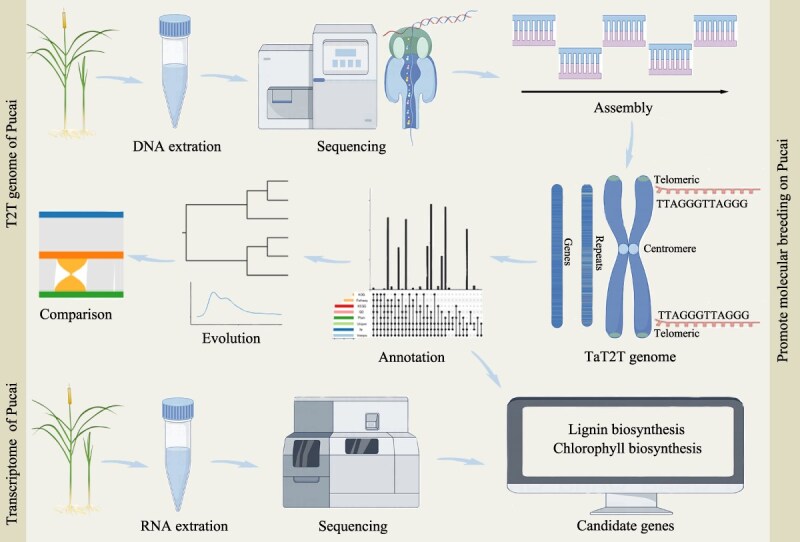
TaT2T genome and transcriptome of Pucai promote molecular breeding on Pucai

In summary, we assembled the TaT2T genome with high quality and the genome size was 207.00 Mb. The low activity of the recent LTR-RT of Pucai and the σ WGD-associated *Ks* peaks from different Poales are related to the slow evolution of Pucai. At the same time, the Typhaceae-WGD event was identified in Typhaceae. Gene families related to lignin, chlorophyll biosynthesis, and disease resistance were greatly expanded. Statistical analysis and annotation of genomic variations were conducted in interspecies and intraspecies of *Typha*, providing important insights into the genetic diversity and agronomic traits of Pucai. Subsequently, the candidate structural genes and TFs that regulate lignin and chlorophyll biosynthesis were identified. The results can provide a theoretical basis for future molecular breeding on Pucai (Fig. 8).

## Material and methods

### Plant material

The Pucai plant cultivated in Huai’an, Jiangsu Province, China, was selected as the material. Leaves exhibiting optimal growth conditions were collected for DNA extraction and genomic analysis. Furthermore, the pseudostem during the autumn harvest was utilized for RNA extraction and subsequent RNA-seq. All samples were rapidly frozen using liquid nitrogen and subsequently stored at −80°C until they were needed for analysis.

### Genomic sequencing

Nanopore sequencing, HiFi sequencing, and Next-generation genome sequencing were performed with reference to Wang [38]. All the sequencing works were carried out by Wuhan Benagen Technology Co., Ltd (China).

### Genome assembly and assessment

The initial assembly of the ONT Ultra-long sequencing data was conducted utilizing NextDenovo v2.5.0, NECAT, and Flye software [74]. For the genome assembly of the PacBio HiFi data, Hifiasm v0.16.1 was employed [75]. The ALLHiC software facilitated the clustering of contig sequences into distinct chromosome groups through a bottom-up hierarchical clustering algorithm [76]. Following the removal of heterozygous sequences, gaps were filled with 100 N to achieve the final chromosome-level genome sequence. HiCExplorer v3.6 was utilized to visualize the interaction intensity and positional relationships between contigs. The *K*-mer anchoring method was implemented to target the centromeric region for genomic error correction across three generations. ONT reads were aligned to pseudochromosomes using minimap2 software. Error correction was performed using Medaka consensus v1.5.0, and the consistent sequence following three generations of error correction was obtained through the medaka stitch [77]. For next-generation error correction, DeepVariant v1.3.0 was employed. Additionally, Winnowmap v.11 was used to gather all reads within 50 bp at the chromosome ends [78]. Genomic assessment was performed with BUSCO software [79]. The telomere sequence 5′-CCCTAAA-3′ and its reverse complement were directly searched [80].

### Genome annotation

The genomic repeat sequences were identified utilizing a *de novo* approach through the RepeatModeler software. Additionally, nonredundant LTR sequences were extracted using the LTR_FINDER and LTR_retriever software tools. Complete LTR elements were used to calculate the LAI value [41]. The methods and processes for genome annotation refer to those previously described [38].

Gene structure prediction in this study uses a combination of transcriptome prediction, homology prediction, and *de novo* prediction [38]. Transcriptome prediction is based on existing sequencing platforms and is divided into second-generation transcriptome prediction and third-generation transcriptome prediction. Homology prediction selects protein sequence files from near-origin species for prediction analysis. *De novo* prediction is based on the genome after masking repetitive sequences for analysis. Using MAKER software, the gene sets predicted by various methods were integrated [81]. Gene function annotation by sequence similarity search and Motif similarity search [38].

Based on the structural features of tRNAs, tRNAscan-SE was used to find tRNA sequences in the genome; rRNA prediction was performed using the rRNA database; and INFERNAL based on the Rfam database was used to find ncRNA sequences in the genome, such as snRNAs, miRNAs, and so on.

### Centromere prediction

Considering the high density of short tandem repeats and the low gene density observed in the centromeric region, TRF software was employed to assess the prevalence of short tandem repeats and gene coverage within the genomic sequence. Additionally, the position of the centromere was predicted utilizing a collinear Circos diagram of the genome [34].

### Genomic evolutionary analysis

The OrthoFinder software was employed for the clustering of gene families utilizing the complete amino acid sequences of the species under investigation, while clusterProfiler was utilized for GO and KEGG analyses. The phylogenetic tree, time of species divergence, and fossil calibration points were constructed referring to those previously described [38]. Significant expansions or contractions were determined at a threshold of *P* < .05, and functional enrichment analyses were conducted on gene families exhibiting significant changes in size. The protein sequence alignments, frequency of synonymous mutation (*Ks*), and positions of similar gene pairs in chromosomes were performed using WGDI [82].

### Genome-wide comparisons

A genome-wide comparison was conducted using MUMmer software. The identification of variants was conducted using SyRI (Synteny and Rearrangement Identifier).

### Transcriptome sequencing

Total RNA was isolated utilizing the RNA Nano 6000 Assay Kit designed for the Bioanalyzer 2100 system. The RNA-Seq libraries were prepared following the protocol established by Illumina and subsequently sequenced using the NovaSeq 6000 platform [83]. The WGCNA analysis was conducted using the WGCNA (v1.69) software.

### Identification of genes associated with lignin and chlorophyll biosynthesis

The TBtools software was employed to perform a BLAST analysis of homologous gene sequences within the TaT2T genome, utilizing an e-value threshold of <10e−5. The accuracy was additionally validated through a comparative analysis of the protein and conserved domains against those in the Pfam database.

### Measurement of lignin and chlorophyll content

The assessment of lignin content was conducted by previously established method [84]. The distribution of lignin within the tissue sections was analyzed using histochemical staining and UV microscopy. Chlorophyll was extracted from fresh plant tissues utilizing 95% ethanol.

## Compliance with ethics requirement

This article does not contain any studies on human or animal subjects.

## Supplementary Material

Web_Material_uhaf079

## Data Availability

The assembled Pucai genome can be downloaded from the NCBI (https://www.ncbi.nlm.nih.gov/) database with the BioProject accession number PRJNA1221093. The transcriptome sequencing data generated in this study have been deposited in the NCBI Sequence Read Archive (SRA) database under accession number PRJNA1221065. The date is available in the figures of the article and its supplementary materials.
