# Supplementary material for: The telomere-to-telomere genome of Pucai ([image]) (Typha angustifolia L.): a distinctive semiaquatic vegetable with lignin and chlorophyll as quality characteristics
Source: Hortic Res. 2025 Mar 11;12(7):uhaf079. doi: 10.1093/hr/uhaf079 (PMC12058305; doi:10.1093/hr/uhaf079)
Supplement: Web_Material_uhaf079 [file web_material_uhaf079.zip › Supplementary Figures.docx]

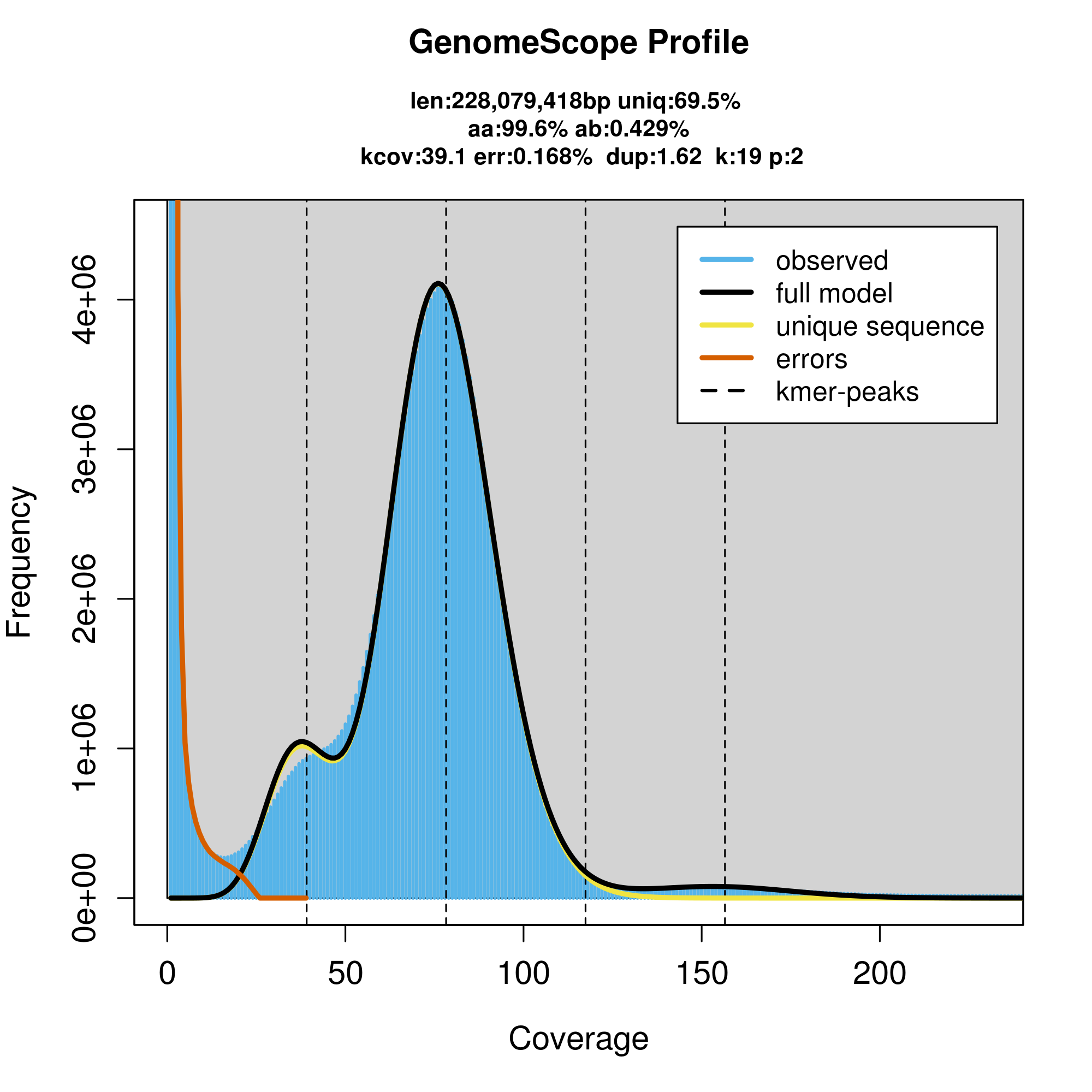


**Figure S1 *K*-mer distribution of sequencing reads of Pucai**


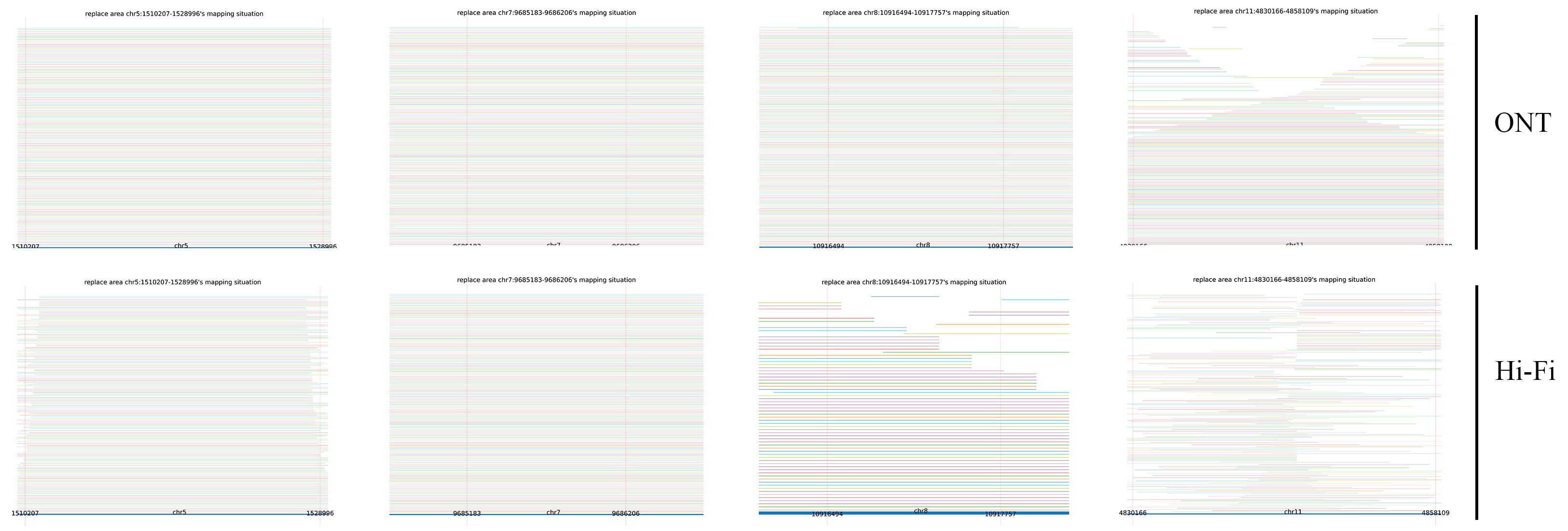


**Figure S2 Mapping locations of reads for gap filling of Pucai**


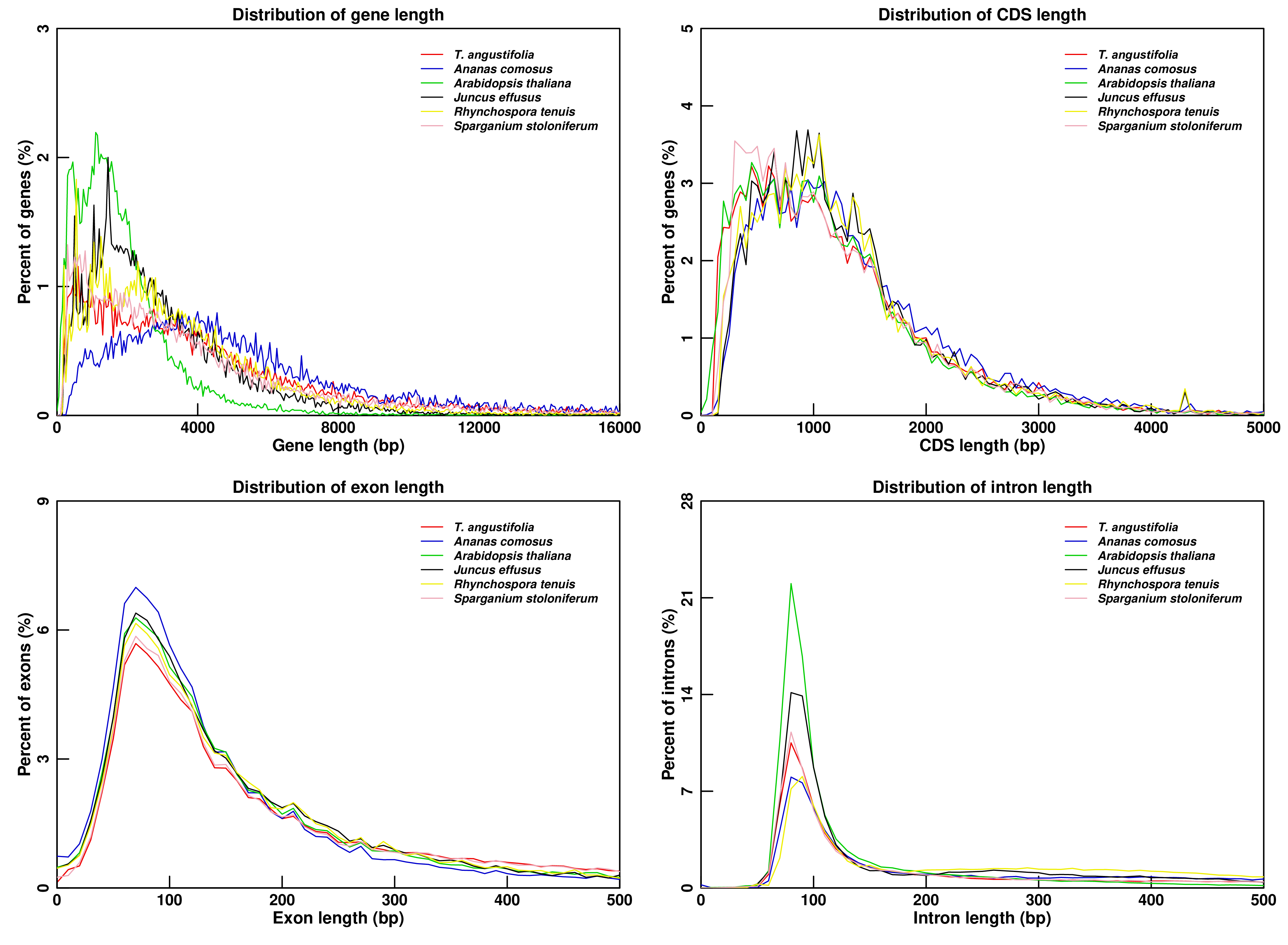


**Figure S3 Comparison of gene lengths between Pucai and 5 species**

**
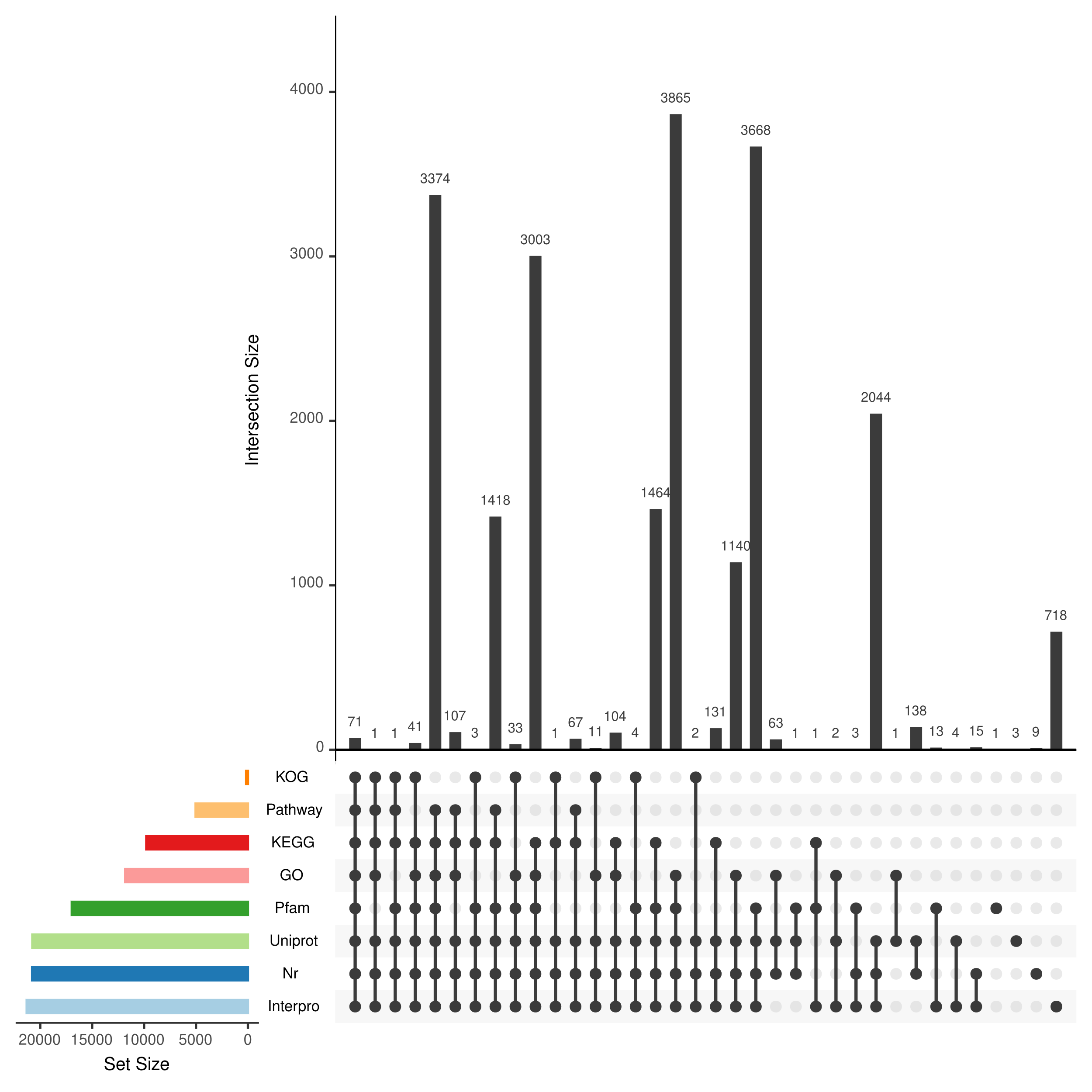
**

**Figure S4 Gene function annotation of TaT2T genome**

**
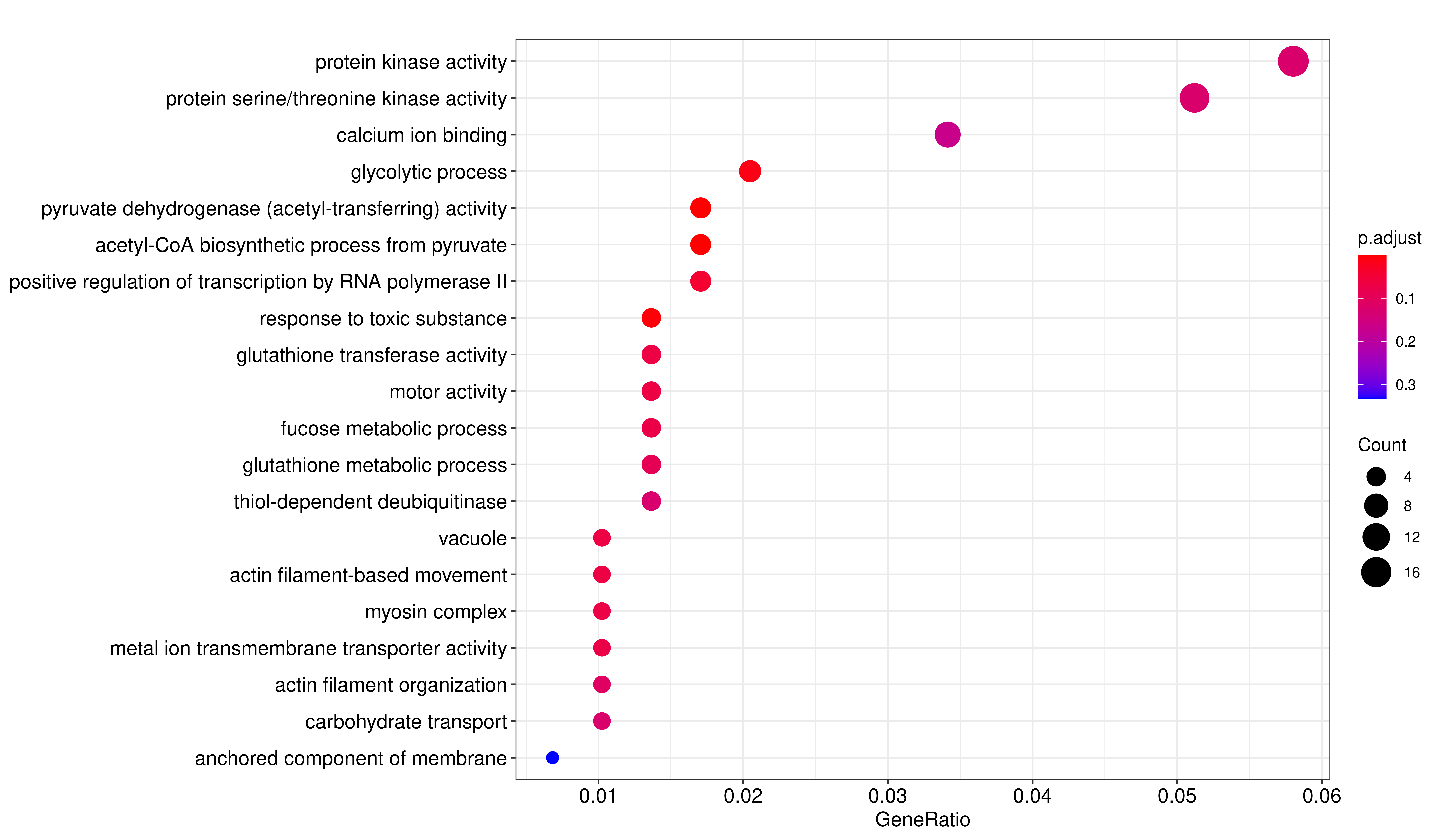
**

**Figure S5 GO enrichment analysis of unique family genes in Pucai**

**
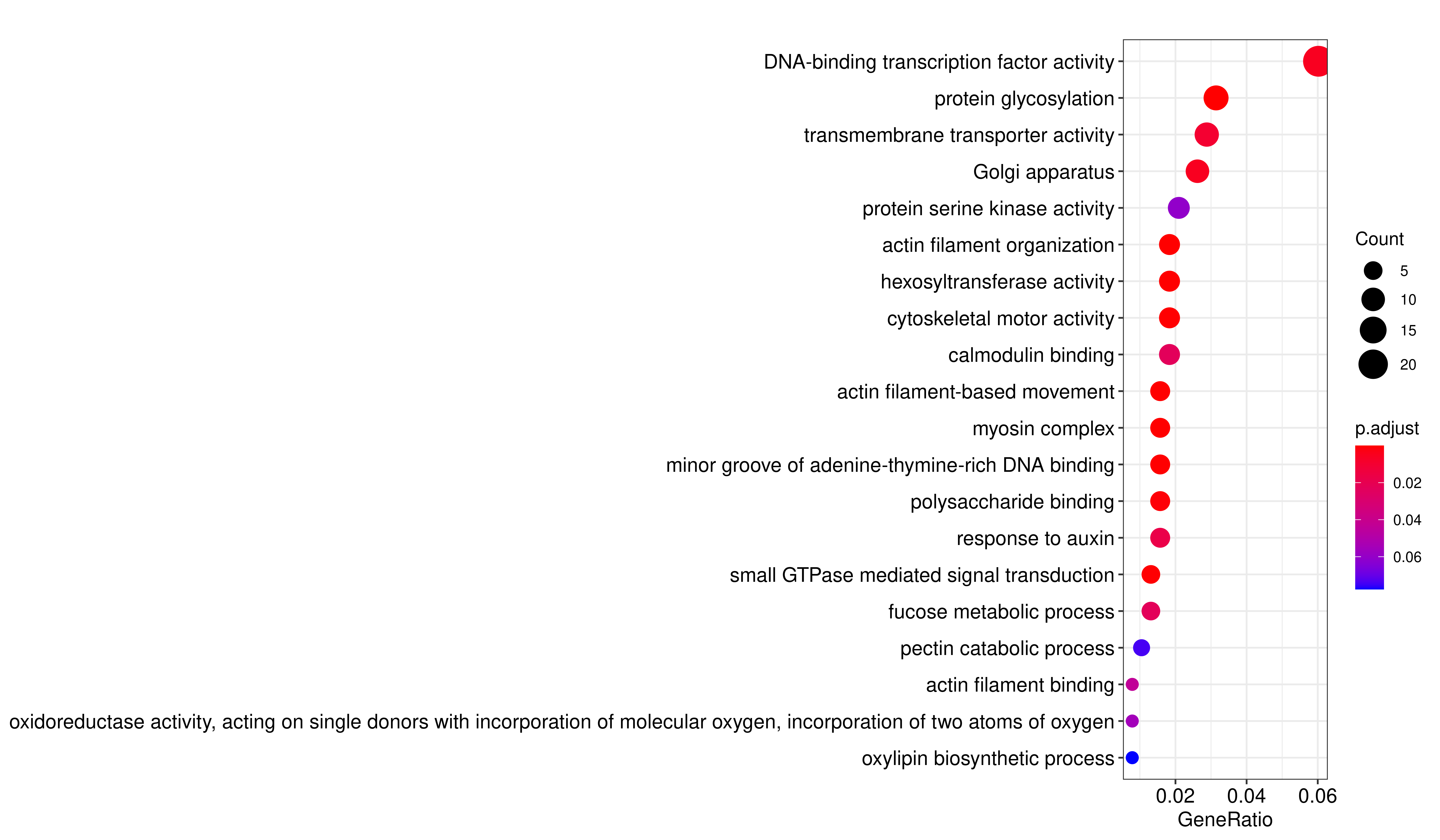
**

**Figure S6 GO enrichment analysis of contraction family genes in Pucai**

**
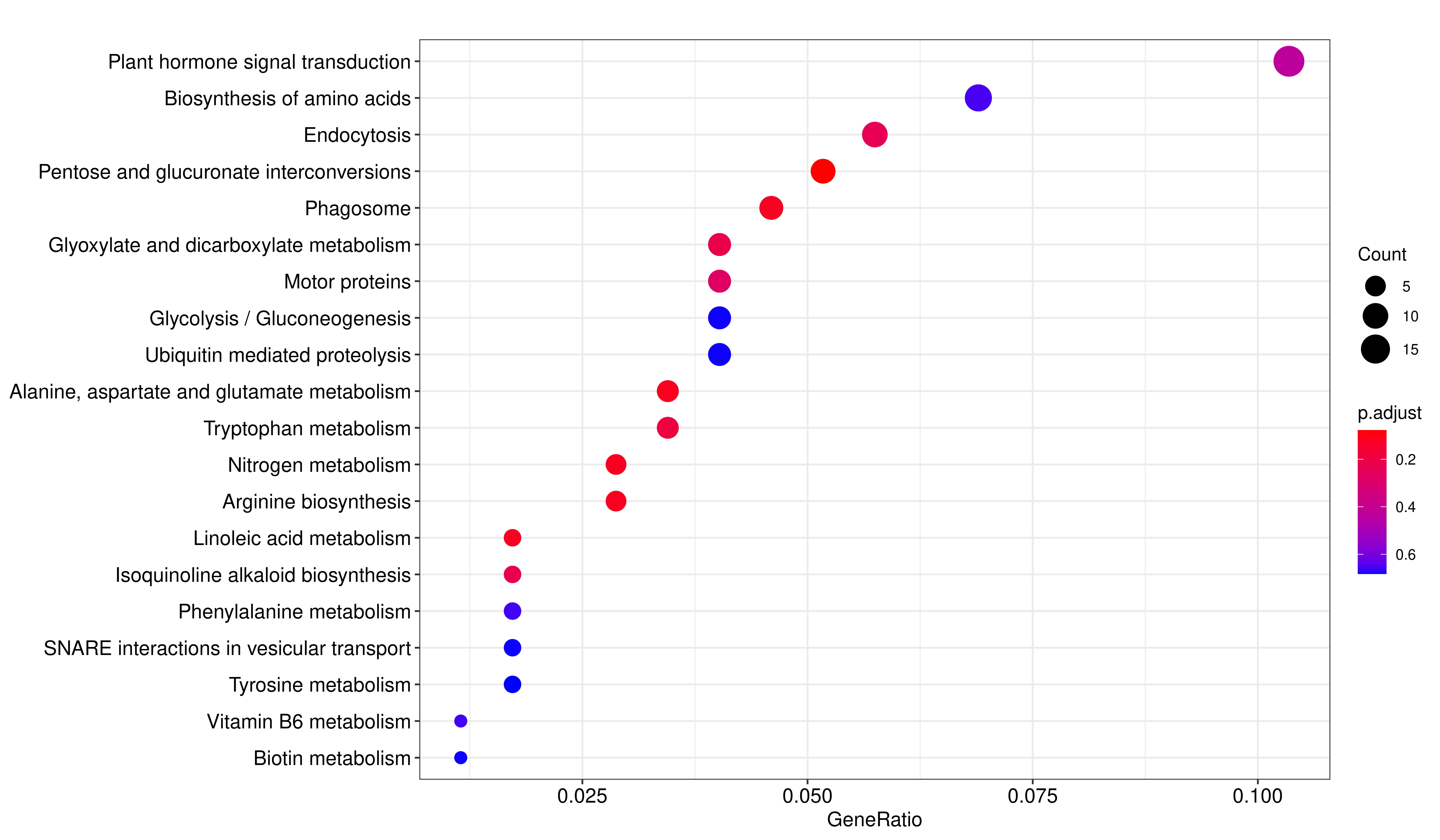
**

**Figure S7 KEGG enrichment analysis of contraction family genes in Pucai**

**
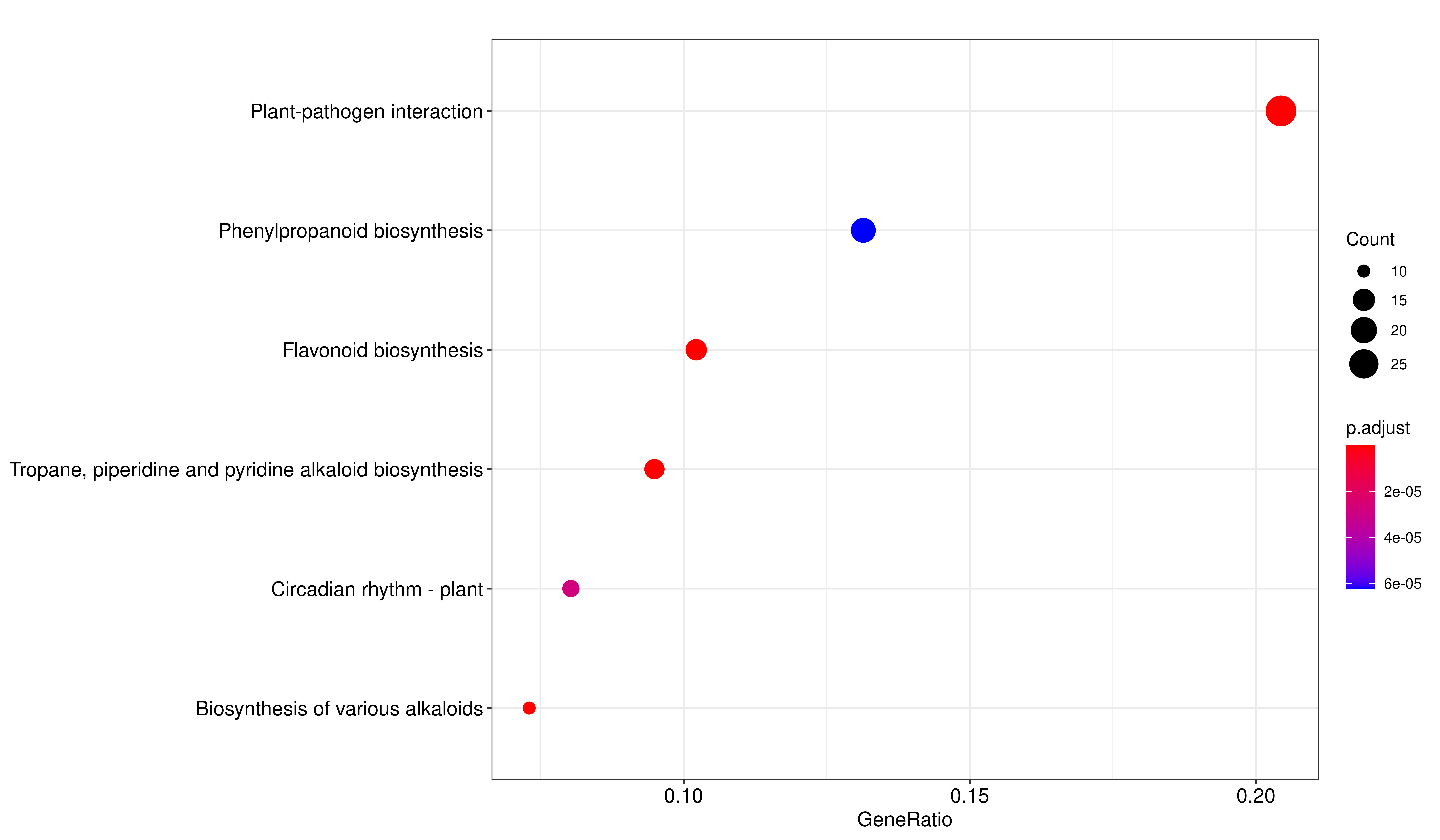
 Figure S8 KEGG enrichment analysis of PD genes in Pucai**

**
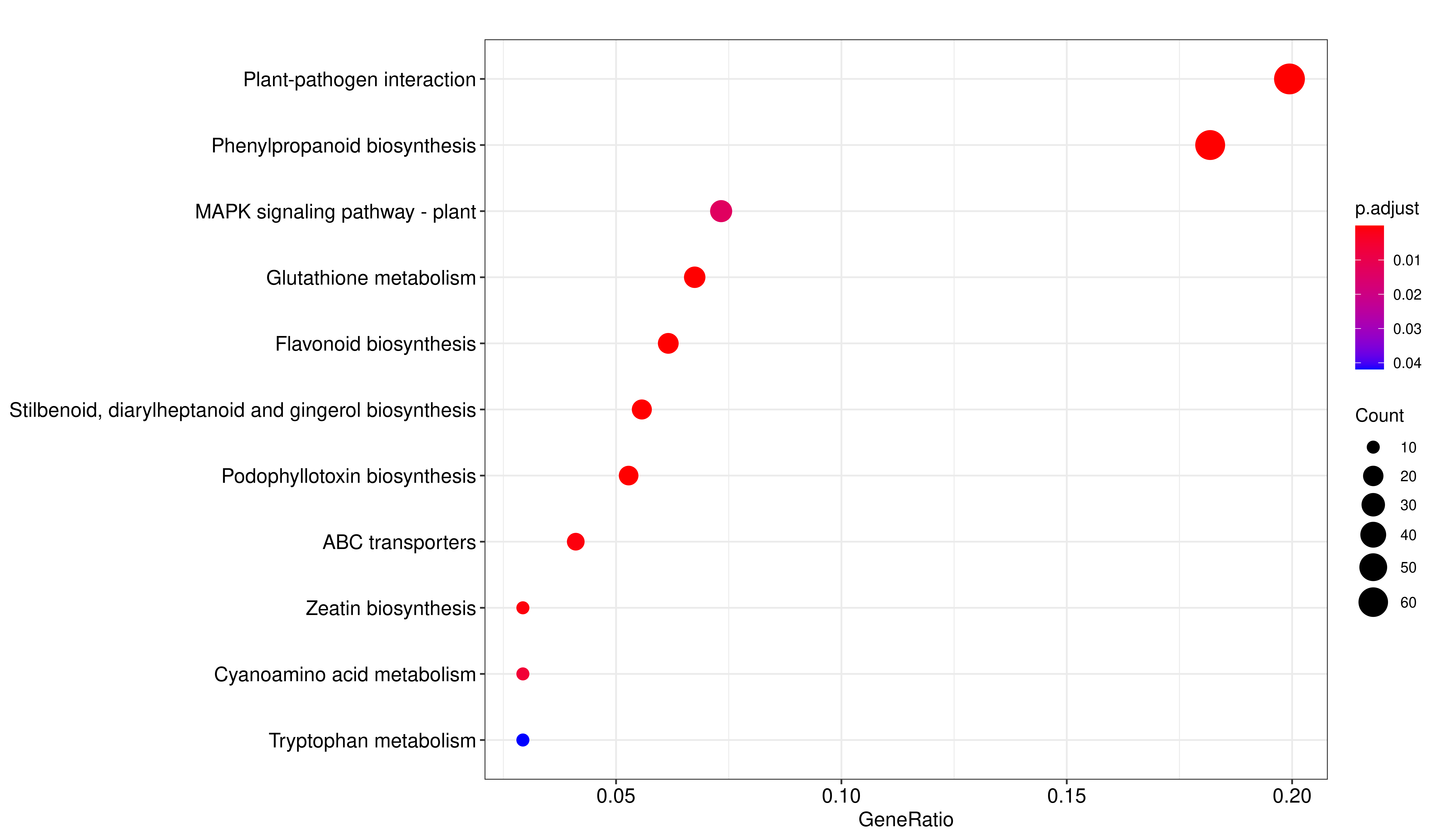
 Figure S9 KEGG enrichment analysis of TD genes in Pucai**

**
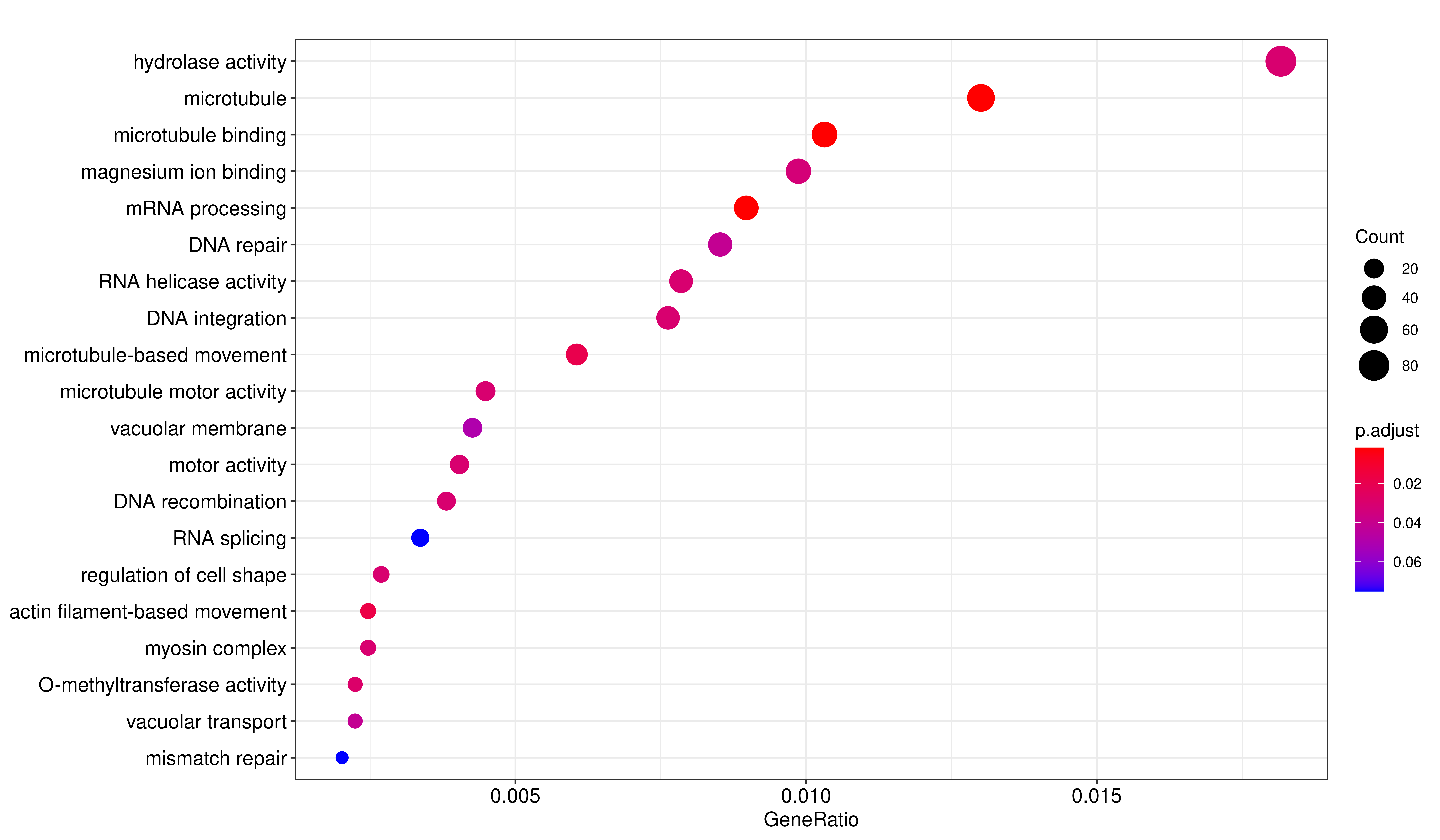
**

**Figure S10 GO enrichment of SVs between species**

**
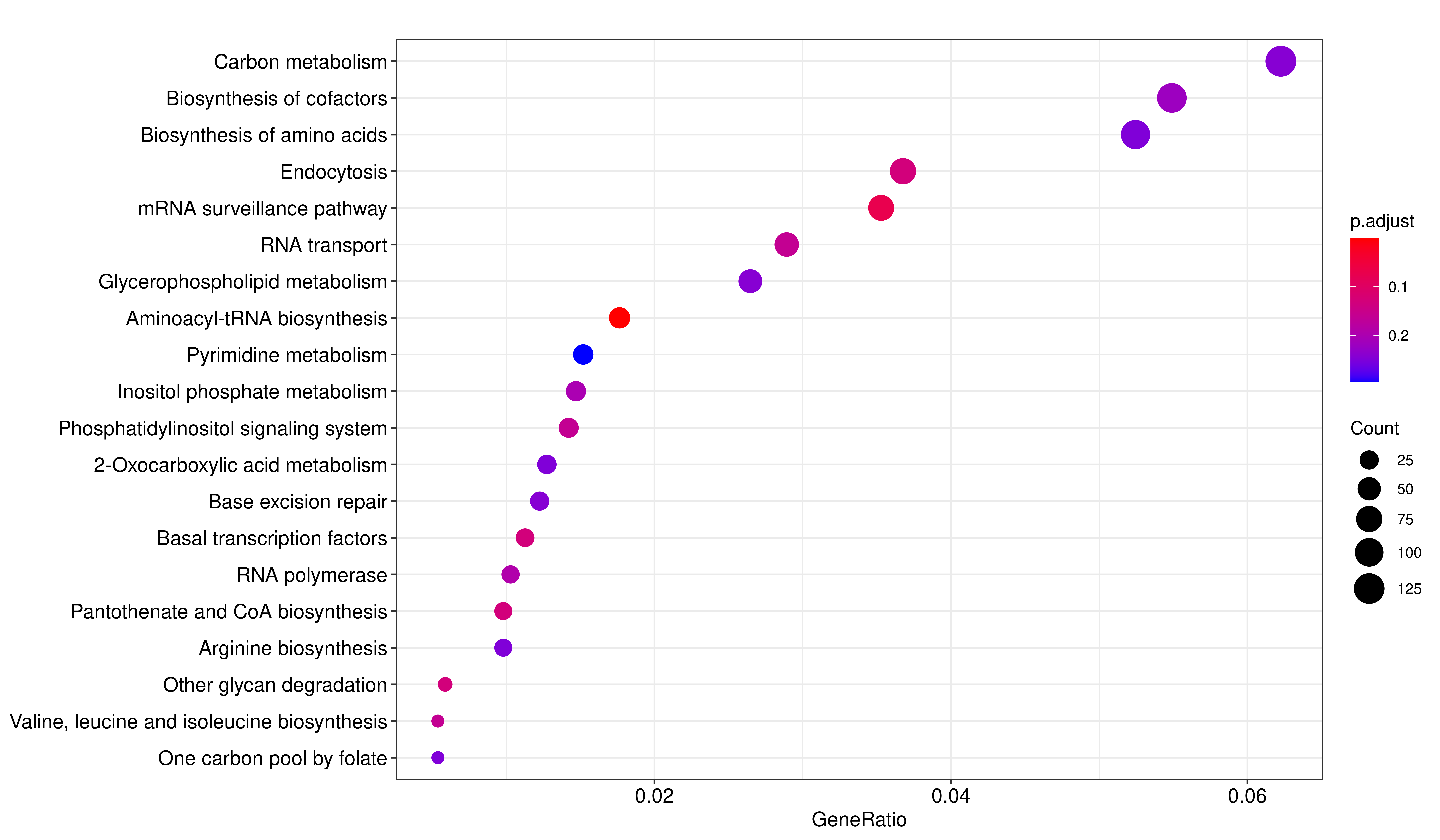
**

**Figure S11 KEGG enrichment of SVs between species**

**
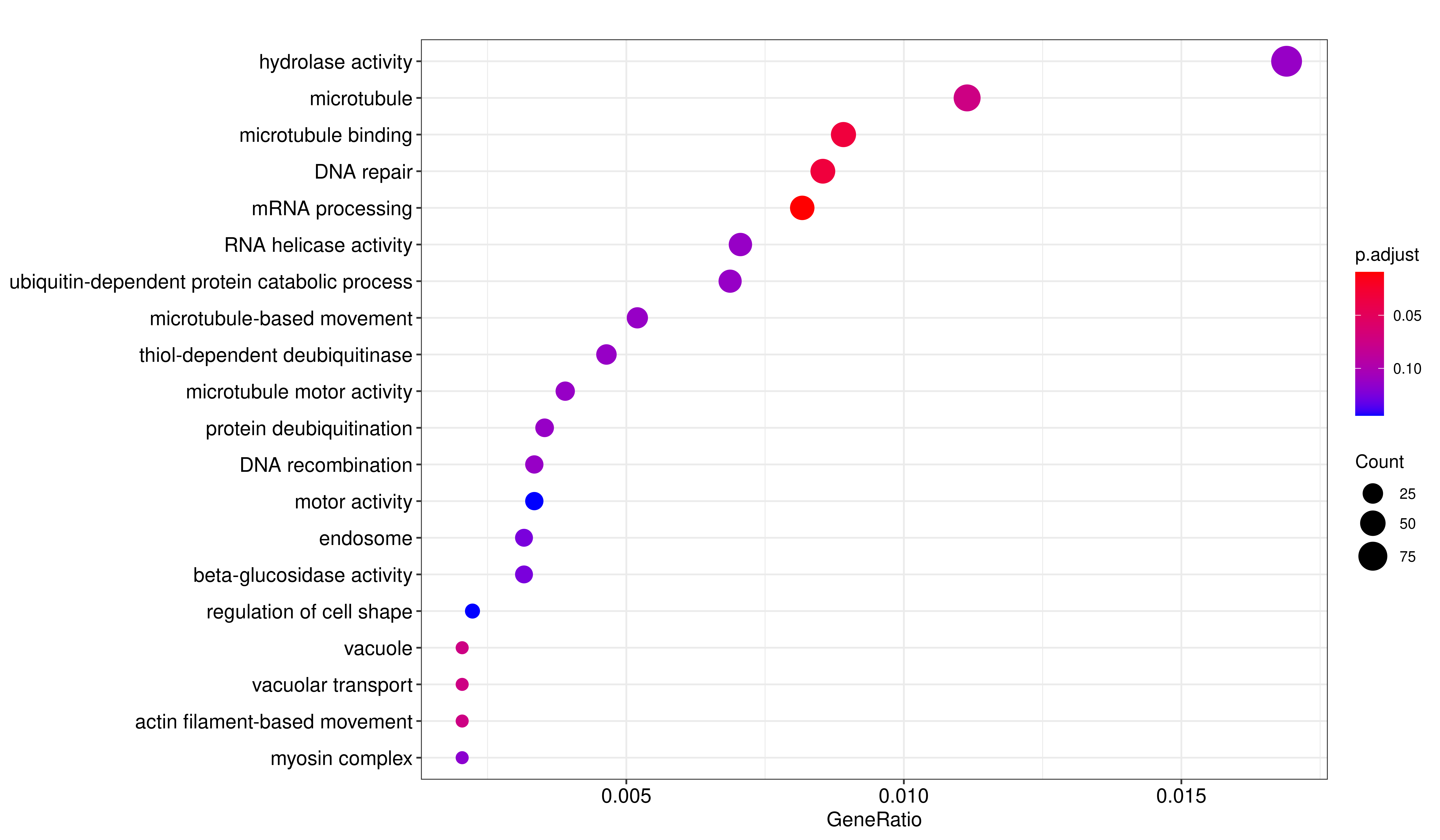
**

**Figure S12 GO enrichment of PAV between species**

**
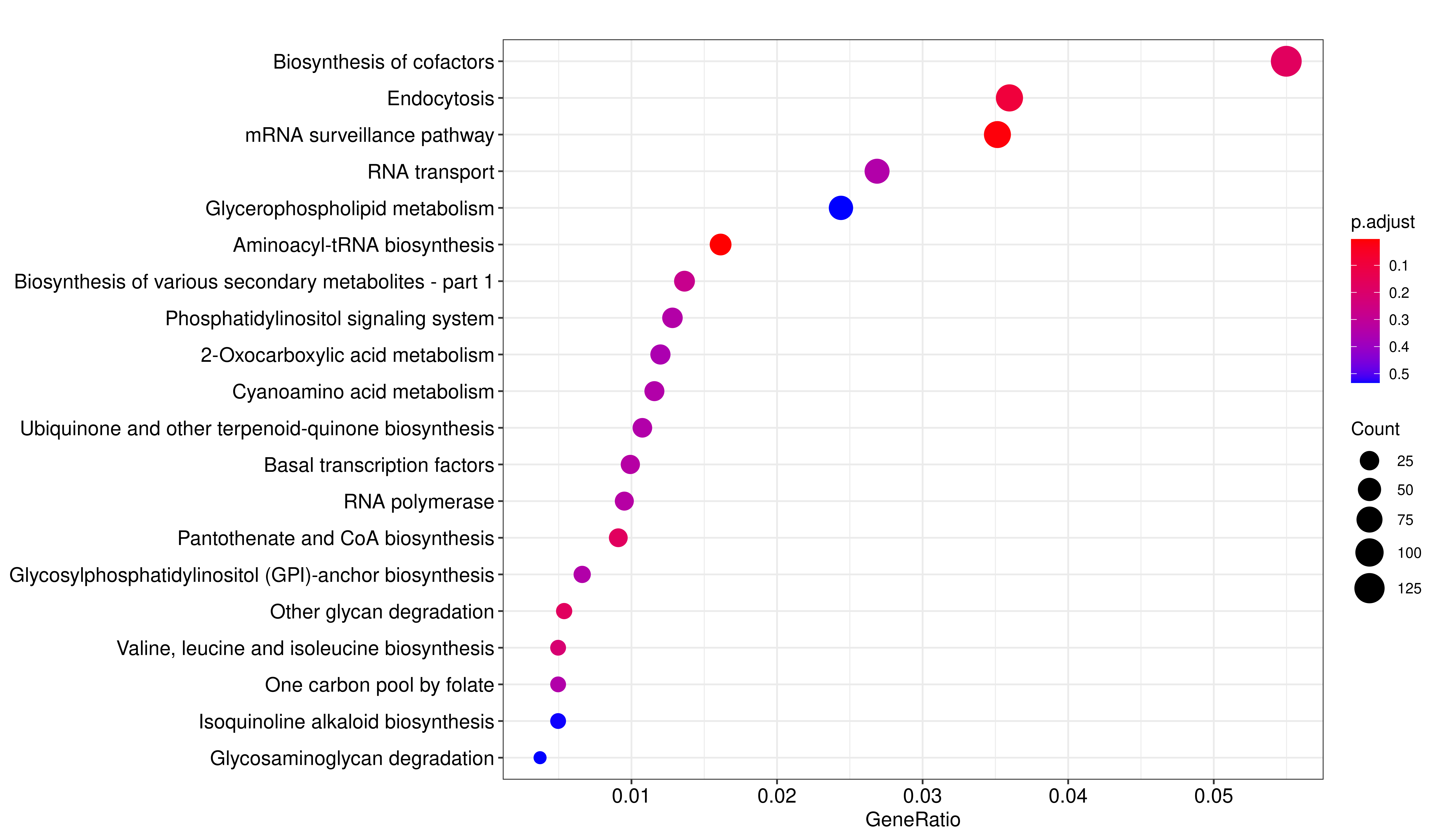
**

**Figure S13 KEGG enrichment of PAV between species**

**
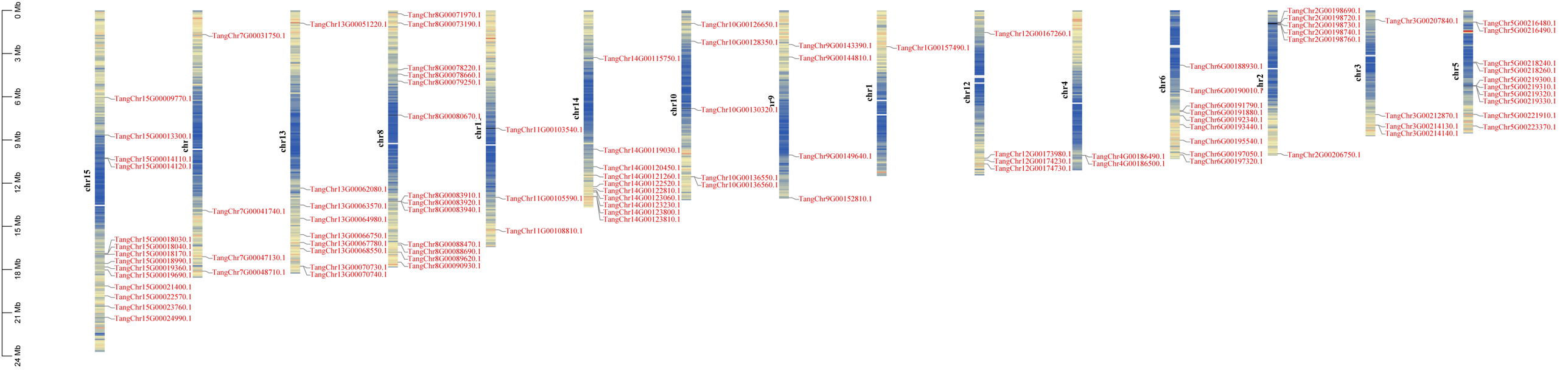
**

**Figure S14 Gene location of lignin biosynthesis genes on chromosomes of Pucai**

**
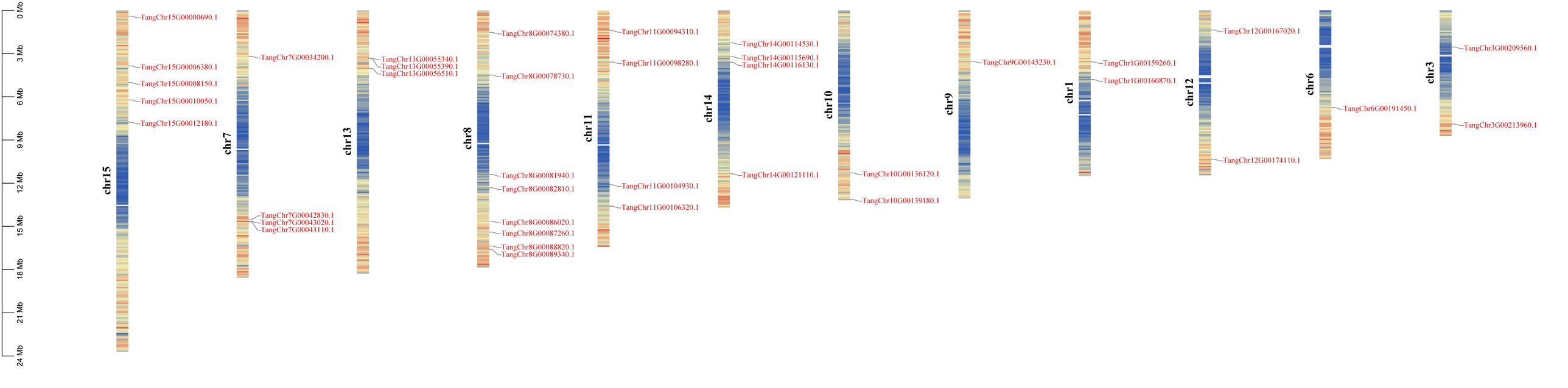
**

**Figure S15 Gene location of chlorophyll biosynthesis genes on chromosomes** **of Pucai**


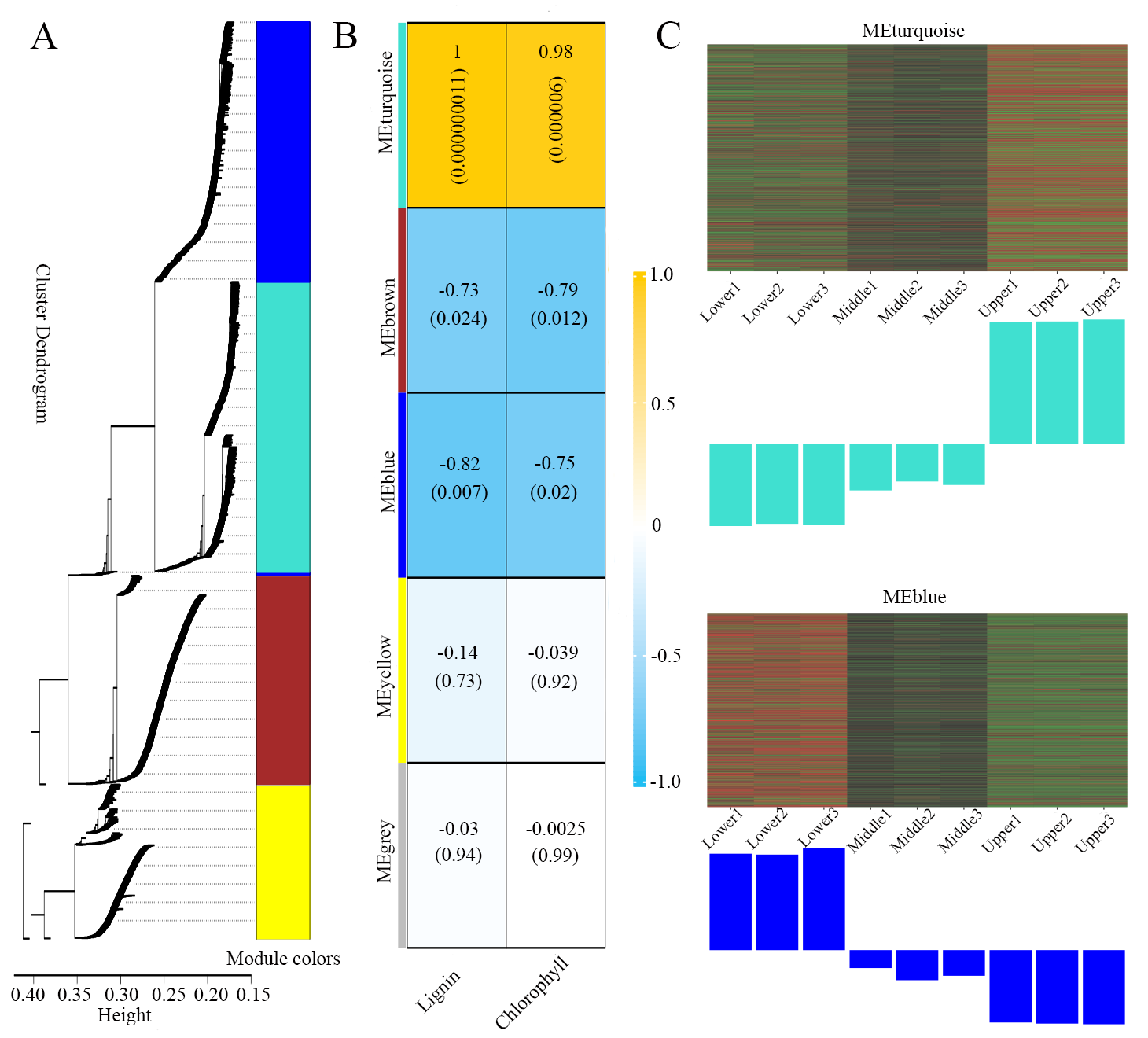


**Figure S16** WGCNA analysis of potential genes that regulate lignin and chlorophyll biosynthesis.

(A): WGCNA dendrogram indicating the expression of 4 different gene modules. (B): Trait and module association analyses. (C): Heatmap of genes in MEturquoise and MEblue module.
